# H2B Mono-ubiquitylation Facilitates Fork Stalling and Recovery during Replication Stress by Coordinating Rad53 Activation and Chromatin Assembly

**DOI:** 10.1371/journal.pgen.1004667

**Published:** 2014-10-02

**Authors:** Chia-Yeh Lin, Meng-Ying Wu, Sophie Gay, Lisette Marjavaara, Mong Sing Lai, Wei-Chun Hsiao, Shih-Hsun Hung, Hsin-Yi Tseng, Duncan Edward Wright, Chen-Yi Wang, Guoo-Shyng W. Hsu, Didier Devys, Andrei Chabes, Cheng-Fu Kao

**Affiliations:** 1 Institute of Cellular and Organismic Biology, Academia Sinica, Nankang, Taipei, Taiwan; 2 Graduate Institute of Nutrition and Food Sciences, Fu-Jen Catholic University, Xinzhuang, New Taipei City, Taiwan; 3 Fondazione Istituto FIRC di Oncologia Molecolare (IFOM), IFOM-IEO Campus, Milan, Italy; 4 Department of Medical Biochemistry and Biophysics, Umeå University, Umeå, Sweden; 5 Institut de Génétique et de Biologie Moléculaire. CNRS UMR 7104, INSERM U 596, Université Louis Pasteur de Strasbourg, Illkirch, CU de Strasbourg, France; MRC Laboratory of Molecular Biology, United Kingdom

## Abstract

The influence of mono-ubiquitylation of histone H2B (H2Bub) on transcription via nucleosome reassembly has been widely documented. Recently, it has also been shown that H2Bub promotes recovery from replication stress; however, the underling molecular mechanism remains unclear. Here, we show that H2B ubiquitylation coordinates activation of the intra-S replication checkpoint and chromatin re-assembly, in order to limit fork progression and DNA damage in the presence of replication stress. In particular, we show that the absence of H2Bub affects replication dynamics (enhanced fork progression and reduced origin firing), leading to γH2A accumulation and increased hydroxyurea sensitivity. Further genetic analysis indicates a role for H2Bub in transducing Rad53 phosphorylation. Concomitantly, we found that a change in replication dynamics is not due to a change in dNTP level, but is mediated by reduced Rad53 activation and destabilization of the RecQ helicase Sgs1 at the fork. Furthermore, we demonstrate that H2Bub facilitates the dissociation of the histone chaperone Asf1 from Rad53, and nucleosome reassembly behind the fork is compromised in cells lacking H2Bub. Taken together, these results indicate that the regulation of H2B ubiquitylation is a key event in the maintenance of genome stability, through coordination of intra-S checkpoint activation, chromatin assembly and replication fork progression.

## Author Summary

Eukaryotic DNA is organized into nucleosomes, which are the fundamental repeating units of chromatin. Coordination of chromatin structure is required for efficient and accurate DNA replication. Aberrant DNA replication results in mutations and chromosome rearrangements that may be associated with human disorders. Therefore, cellular surveillance mechanisms have evolved to counteract potential threats to DNA replication. These mechanisms include checkpoints and specialized enzymatic activities that prevent the replication and segregation of defective DNA molecules. We employed a genome-wide approach to investigate how chromatin structure affects DNA replication under stress. We report that coordination of chromatin assembly and checkpoint activity by a histone modification, H2B ubiquitylation (H2Bub), is critical for the cell response to HU-induced replication stress. In cells with a mutation that abolishes H2Bub, replication progression is enhanced, and the forks are more susceptible to damage by environmental insults. The replication proteins on replicating DNA are akin to a train on the tracks, and movement of this train is carefully controlled. Our data indicate that H2Bub helps organize DNA in the nuclei during DNA replication; this process plays a similar role to the brakes on a train, serving to slow down replication, and maintaining stable progression of replication under environmental stress.

## Introduction

Recent evidence suggests that histone modifications can affect DNA replication, under both normal or stressed conditions, through effects on nucleosome dynamics and protein recruitment [1]–[3]. One such modification is acetylation of nascent histone H3 at lysine 56 (H3K56Ac), which is regulated by the Asf1 histone chaperone and the Rtt109 acetyltransferase during the cell cycle [4], [5]. Regulation of this modification is important for DNA replication, as failure to deacetylate H3K56Ac results in impaired S phase progression [6], sensitivity to replication stress [7], and spontaneous DNA damage [6]. H3K56Ac appears to facilitate nucleosome reassembly on daughter strands during S phase [8]. Acetylation of the N terminal lysines of histone H3 by Gcn5 also contributes to nucleosome assembly during DNA replication [9]. These findings suggest that replication-coupled nucleosome assembly may impact on both fork progression and the stability of stalled forks [1], [3]. A second histone, H2B, is mono-ubiquitylated at lysine 123 (K123, K120 in human) by the E2 enzyme Rad6 and the E3 enzyme Bre1 in *Saccharomyces cerevisiae* [10]–[13]. Mono-ubiquitylation of H2B (H2Bub) is best characterized in terms of its effects on transcriptional regulation in budding yeast [14], [15], which are mediated through downstream methylation of lysines 4 and 79 of H3 [16]–[19]. In addition, H2Bub has been demonstrated to affect transcription independently of its regulation of H3 methylation [20], [21]. H2Bub enhances passage of RNA Polymerase II during transcription elongation by mediating nucleosome reassembly in both yeast and human [20], [22], [23]. Furthermore, H2Bub may also affect transcription and DNA repair through influencing chromatin structure [24], [25]. It has been suggested that H2Bub mediates homologous recombination repair at DNA double-strand break (DSB) sites through relaxing chromatin structure in human cells [26], [27]. H2Bub has also been shown to maintain replication fork stability by promoting replication-associated nucleosome formation in budding yeast, independently of its role in regulating H3K4 and K79 methylation [28].

During S phase, replication fork progression can be impaired by low dNTP pools or by DNA damage. Under these conditions, a sensor-response system activates the DNA replication (intra-S phase) checkpoint, which prevents fork collapse while controlling origin firing [29], [30]. The mechanism by which the intra-S checkpoint is activated is still not yet fully understood. It is hypothesized that decoupling between polymerase and helicase leads to single strand DNA accumulation and activation of the kinases Mec1/ATR and their downstream effector, Rad53 [30], [31]. Once a stalled fork has been stabilized by activation of the intra-S checkpoint, the damaged fork can resume DNA synthesis. The RecQ helicase Sgs1 is recruited to the stalled fork, where it facilitates its re-initiation through a mechanism involving the recombination repair pathway [32]. Sgs1 also facilitates the phosphorylation of Rad53 (possibly through direct physical interaction), and this process is redundant with the DNA damage checkpoint proteins Rad24 and Esc2 [33].

Activation of the intra-S phase checkpoint affects DNA synthesis by altering both the rate of replication fork progression and the rate of DNA replication initiation events [34]. For instance, a recent report suggests that Mec1 promotes chromatin accessibility at or ahead of replication forks via a mechanism independent of its checkpoint role [35]. The authors argue that a chromatin regulatory process may serve as a means of restricting fork progression, in order to control and stabilize fork progression under replication stress. However, the mechanisms through which chromatin structure regulates replication progression are still poorly understood.

In the current study, we used BrdU IP-chip to examine genome-wide DNA synthesis incorporation in wild type and H2Bub-deficient cells in the presence of hydroxyurea (HU). We demonstrate that newly-synthesized DNA in cells lacking H2Bub displays a broader distribution and enrichment at origin-distal regions; these findings suggest faster replication fork progression in the mutant. Surprisingly, this phenomenon is independent of DNA damage-induced dNTPs, and is accompanied by delayed Rad53 activation and defective chromatin assembly. All of these effects contribute to replication fork instability and reduced cell viability under replication stress. Our data indicate that H2Bub is one of the limiting factors that regulate replication fork progression, and maintain fork stability in the presence of HU-induced stress.

## Results

### H2B mono-ubiquitylation regulates fork progression in HU

**Figure 1:**
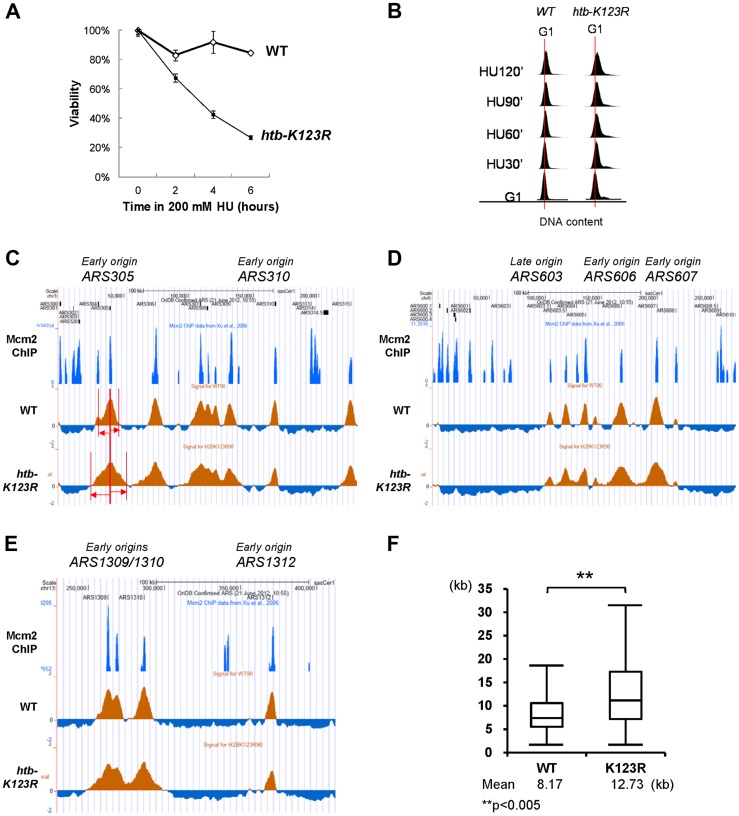
H2Bub regulates fork stalling in HU. (A) The response of WT (CFK1204) and *htb-K123R* (CFK1231) cells to acute doses of HU. Log-phase cells were treated with 0.2M HU for the indicated times, and dilutions were subsequently spread onto YPD plates. The plates were incubated at 30°C for 2–3 days and viability was estimated based on colony forming units (CFU). Viability was normalized to 0 min of HU treatment, which was set as 100%. (B) Flow cytometry was used to analyze the cell cycle progression of WT (CFK1204) and *htb-K123R* (CFK1231) cells in the presence of 0.2M HU for 120 minutes after release from α-factor-induced G1 arrest. DNA content is visualized by propidium iodide incorporation. (C–E) Replication profiles of replication origins: (C) *ARS305* and *ARS310*, (D) *ARS603*, *ARS606*, and *ARS607*, and (E) *ARS1309/1310* and *ARS1312*, in WT (CFK1419) and *htb-K123R* (CFK1421) cells. Cells were synchronized in G1 with α-factor, and then released into media containing 0.2M HU and 200 µg/ml BrdU for 90 minutes. After DNA extraction and fragmentation, BrdU-labeled DNA was immunoprecipitated and hybridized on high-resolution oligonucleotide tiling arrays. Orange histogram bars (BrdU) on the y axis represent the average signal ratio on a log2 scale of loci along the reported regions. Positions of ARS elements are identified by Mcm2 occupancy [72]. (F) The graph depicts the distribution of BrdU track lengths in WT (CFK1419) and *htb-K123R* (CFK1421) cells. Box and whiskers indicate the minimum, maximum, and 25–75 percentiles, respectively. Mean BrdU tracks lengths are indicated in kb. Asterisks indicate the P-value of the statistical test (Mann–Whitney rank sum t-test, ** P-value<0.005).

The presence of 200 mM HU increased lethality in mutant cells lacking H2Bub (*htb-K123R* mutant) (Fig. 1A), confirming the previously-hypothesized role of H2Bub in maintaining fork stability [28]. High doses of HU, an inhibitor of the ribonucleotide reductase, leads to a strong decrease in dNTP pools, that in turn leads to a decrease in replication speed and intra-S checkpoint activation [36], [37]. In order to investigate the mechanisms by which H2Bub sustains cell viability during replication stress, we examined genome-wide origin firing and replication fork progression under HU in wild type and in *htb-K123R* cells. This was achieved by performing BrdU immunoprecipitation followed by hybridization on a high density oligonucleotide array. Wild-type (WT) or H2Bub-deficient mutant (*htb-K123R*) cells were pre-synchronized in G1 with α-factor (Fig. 1B) and then released into fresh media containing HU and BrdU. Under such conditions, BrdU is incorporated at active origins (such as *ARS305* and *ARS607*), and BrdU track length correlates with the replication fork progression. Positions of *ARS* elements were identified by Mcm2 occupancy [38]; therefore, this assay can be used to monitor origin usage and replication fork progression on a genomic scale [37].

An unexpected finding was that the BrdU track lengths at most origins were significantly longer in *htb-K123R* cells (average 12.73 kb) than in WT cells (average 8.17 kb; Fig. 1C–F and Fig. S1), indicating extended progression of replication forks. This finding was corroborated by the observation that DNA content in the mutant was greater than in WT, as evidenced by FACS (Fig. 1B). In addition, despite the semi-quantitative aspect of this technique, BrdU incorporation peaks were clearly reduced at the majority of firing origins in the mutant. This may be indicative of a decrease in origin firing. Taken together, these results suggest that replication fork stalling is reduced in the *htb-K123R* mutant during HU-induced stress, and this may lead to fork destabilization.

### H2Bub-mediated fork stalling is independent of Dun1-mediated dNTP regulation

It was previously reported that yeast cells with persistently-enlarged dNTP pools are prone to DNA damage [39], and exhibit enhanced fork progression [37], [40] under replication stress. Thus, it is possible that the enhanced HU sensitivity and increased fork progression in *htb-K123R* cells may be a consequence of enlarged dNTP pools; this in turn may be a direct consequence of (i) increased transcription of ribonucleotide reductase (RNR) genes or (ii) spontaneous DNA damage, or otherwise via an indirect mechanism that stimulates ribonucleotide production. To test this hypothesis, we directly examined the size of dNTP pools in *htb-K123R* cells. We observed that the dNTP concentration in *htb-K123R* cells is ∼40% greater than that of WT cells (shown for four biological replicates in Fig. S2).

**Figure 2:**
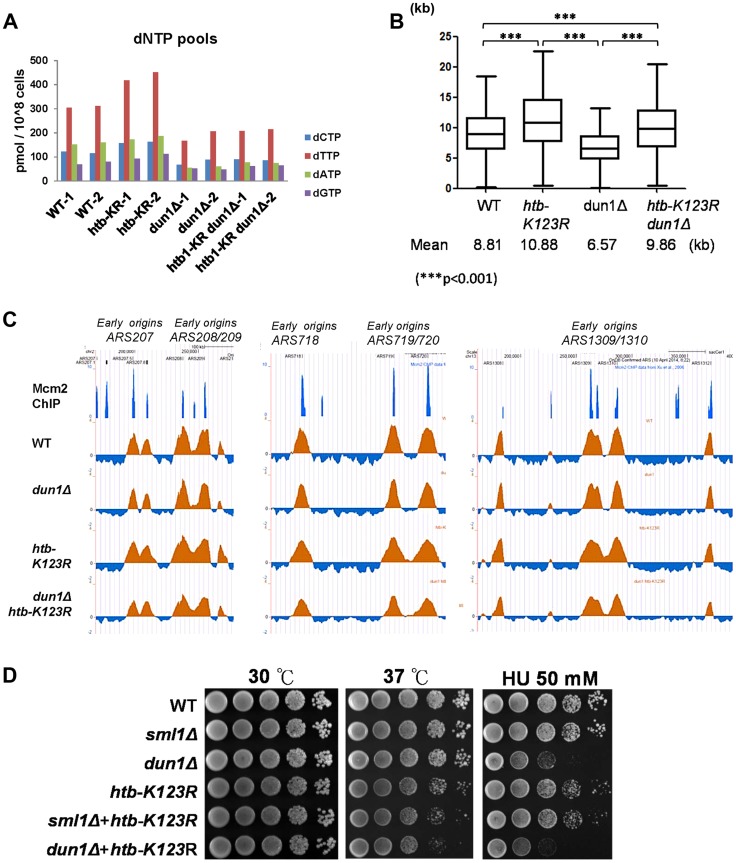
H2Bub-mediated fork stalling is independent of dNTP pool size. The size of the dNTP pools in exponentially-growing cultures of WT (CFK1419), *htb-K123R* (CFK1421), *dun1Δ* (YCL023), and *dun1Δ htb-K123R* (YCL025) cells in YPD media. Two independent isogenic strains of each genotype were analyzed. (B) Graph depicting the distribution of BrdU track lengths in WT (CFK1419), *htb-K123R* (CFK1421), *dun1Δ* (YCL023), and *dun1Δ htb-K123R* (YCL025) mutants, as shown in Fig. 1F. (C) Replication profiles of the replication origins *ARS207*, *ARS208/209*, *ARS718*, *ARS719/720*, and *ARS1309/1310* in WT (CFK1419), *htb-K123R* (CFK1421), *dun1Δ* (YCL023), and *dun1Δ htb-K123R* (YCL025) mutants. The BrdU histogram was analyzed as described in Fig. 1C–E. (D) Temperature sensitivity and HU resistance of the indicated genotypes (WT (CFK1204), *sml1Δ* (CFK1481), *dun1Δ* (YMW069), *htb-K123R* (CFK1231), and *htb-K123R* in combination with *sml1Δ* (CFK1482) or *dun1Δ* (YMW072)). Log-phase cells were serially diluted and spotted onto YPD plates with or without HU, and incubated at 30°C or 37°C for 2–3 days.

The cellular concentration of dNTP pools is regulated by the Rad53-Dun1 pathway during both normal and perturbed cell cycles, through multiple mechanisms [41], [42]. Deletion of *DUN1* stabilizes the RNR inhibitor Sml1 and decreases the size of the cellular dNTP pool, while deletion of *SML1* increases pool size [43]. To further investigate whether the effect of H2Bub on fork stalling during replication stress is dependent on the concentration of dNTP pools, we deleted the *DUN1* gene from WT and *htb-K123R* cells. Notably, the dNTP pools of both *dun1Δ* and *dun1Δ htb-K123R* were ∼50% the size of those in WT cells (Fig. 2A). Since deletion of *DUN1* suppressed the increase of dNTP in H2Bub mutants, we can conclude that the increase of dNTP level in H2Bub mutants is mediated by Dun1.We next performed BrdU-IP chip experiments to determine BrdU track length in these cells. As expected, the BrdU track length of the *dun1Δ* cells was significantly shorter (6.57 kb; Fig. 2B & C) than that in WT cells, probably due to the reduced concentration of dNTP [37], [39]. Surprisingly, fork progression in *dun1Δ htb-K123R* was significantly faster than in WT cells (9.86 kb *vs.* 8.81 kb; Fig. 2B & C), despite the reduced size of the dNTP pool (Fig. 2A); this finding indicates that the increase in fork progression and instability in this mutant does not arise solely from the increase in the dNTP pool. In addition, we observed that deletion of *DUN1*, but not *SML1*, increased the sensitivity of *htb-K123R* cells to chronic HU exposure (Fig. 2D). Therefore, we conclude that H2Bub has a role in controlling fork progression and cell survival in response to replication stress, which is independent of the Dun1-mediated regulation of ribonucleotide production.

### H2B ubiquitylation sets replication dynamics and replication fork integrity under HU stress

**Figure 3:**
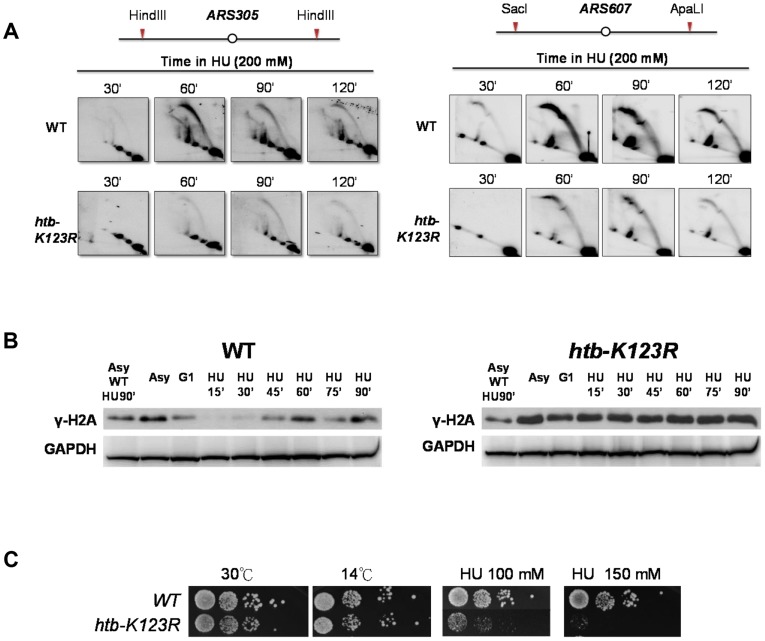
H2Bub preserves replication fork stability under HU stress. (A) Analysis of replication intermediates (RIs) at *ARS305* and *ARS607* in WT (CFK1204) and *htb-K123R* (CFK1231) mutants. Cells were synchronized at G1 phase and released into media containing 200 mM HU for 120 minutes. DNA was prepared from cells collected at the indicated times, cut with HindIII (*ARS305*) or SacI and ApaL1 (*ARS607*), and analyzed by 2D gel using the *ARS305* or *ARS607* probe, as described in the Materials and Methods. (B) Accumulation of damaged DNA in H2Bub-depleted cells. WT (CFK1204) and *htb-K123R* (CFK1231) cells were arrested in G1 and released into fresh media containing 0.2M HU for 90 minutes at 30°C. Whole cell lysates were prepared at the indicated time points, and analyzed by Western blot using antibodies against γ-H2A, a marker of DNA damage. G6PDH was used as a loading control. Asy: Asynchronized cells. (C) Cells lacking H2Bub are more sensitive to replication stress. Ten-fold serial dilutions of yeast cells (WT (CFK1204) and *htb-K123R* (CFK1231)) were spotted onto nonselective YPD plates under different temperatures or YPD containing 100 or 150 mM HU for a period of several days.

Origin firing and fork progression have been reported to be strongly co-regulated by cells in order to ensure normal completion of replication. In particular, an increase in replication speed leads to a decrease in origin firing [37]. Our BrdU immunoprecipitation and chip hybridization data are consistent with this reported tendency (Fig. 1). However, since this technique is only partially quantitative, we decided to confirm this observation using two-dimensional (2D) gel analysis (Fig. 3A). Replication intermediates migrate differently depending on their molecular weight and sterical conformation. In particular, the bubble arc reflects origin firing. Interestingly, both WT and H2Bub mutant exhibit similar replication kinetics at two early origins (*ARS305* and *ARS607*); replication intermediates appear one hour after alpha factor release in agreement with origin firing, and start to decrease after 2 hours, reflecting fork progression outside of the restriction fragment. However, we observed a strong reduction of replication intermediates in the H2Bub mutant compared to WT, likely due to a decrease in origin efficiency.

To further delineate the role of H2Bub in origin firing, we measured incorporation of BrdU into chromatin, using BrdU-IP combined with quantitative-PCR. This experiment was performed at 20°C to slow down DNA replication. We found that replication efficiency at *ARS305* and *ARS607* was much lower in mutant than in WT cells. We did not observe DNA synthesis at a telomeric region (TEL VI) in either WT or the mutant, owing to the late onset of DNA replication at telomere. These data strongly suggest that origin firing is inefficient in cells lacking H2Bub (Fig. S3).

We subsequently hypothesized that the change in replication dynamics in cells lacking H2Bub may affect the integrity of the fork, as previously observed [37]. In particular, γH2A accumulation in the H2Bub mutant confirmed the accumulation of damage in the absence of H2B ubiquitylation (Fig. 3B). This accumulation may explain the hypersensitivity to hydroxyurea that we and others [28] have observed (Fig. 3C).

### The Bre1-H2Bub pathway genetically interacts with components of the intra-S phase checkpoint

**Figure 4:**
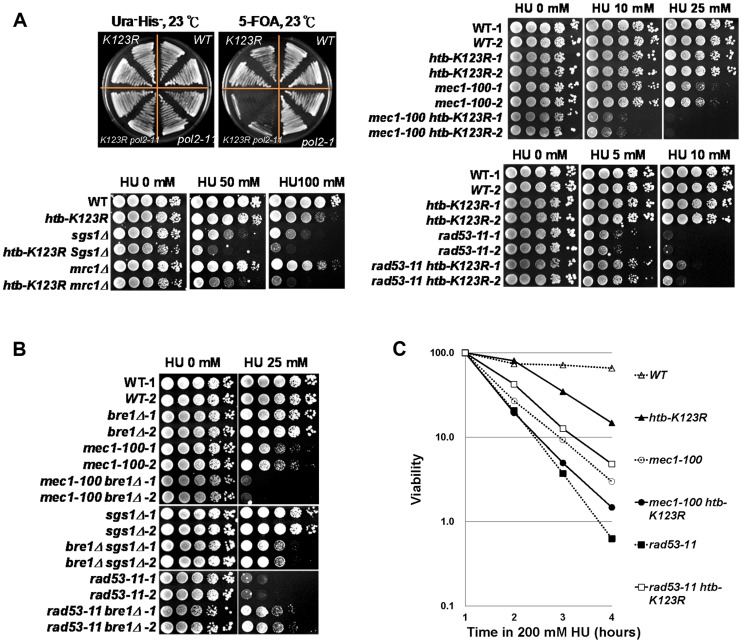
The Bre1-H2Bub pathway genetically interacts with components of the intra-S-phase checkpoint. (A) H2Bub functions in parallel with DNA polymerase (*pol2-11*) and intra-S-phase checkpoint cascades (Mec1, Sgs1, and Mrc1). WT and *pol2-11* cells carrying *HTB1* or the *htb1-K123R* allele on a HIS3 vector were transformed with *HTB1* on a *URA3* vector. The strains containing both *URA3* and *HIS3* (CFK2000, CFK2002, CFK2004, and CFK2006) were streaked onto 5-FOA plates to select for cells lacking H2Bub (*htb1-K123R*). Ten-fold serial dilutions of the indicated strains were spotted onto YPD plates in the absence or presence of different doses of HU at 30°C (WT (CFK1204, CFK2352, and CFK2414), *htb-K123R* (CFK1231 and CFK2416), *mec1-100* (CFK2346), *sgs1Δ* (CFK1447), *mrc1Δ* (CFK1444), *rad53-11* (CFK2347), and double mutants (CFK2356, CFK1453, CFK1450, and CFK2358)). (B) The H2B ubiquitin E3 ligase, Bre1, functions in parallel with intra-S-phase checkpoints under HU stress. Ten-fold serial dilutions of the indicated strains (WT (CFK2351), *bre1Δ* (YMW093), *mec1-100* (CFK2346), *mec1-100 bre1Δ* (YMW095), *sgs1Δ* (CFK2371), *bre1Δ sgs1Δ* (CFK2373), *rad53-11* (CFK2347), and *rad53-11 bre1Δ* (CFK2378)) were spotted onto YPD plates with and without HU as described in (A). (C) The response of double mutants of *htb-K123R* and *mec1-100* or *rad53-11* to acute exposure to HU. Logarithmically-growing cells were treated with 0.2M HU as described in Fig. 1A.

Stalled forks are detected by the intra-S phase checkpoint [30]. We reasoned that the instability of replication forks in *htb-K123R* cells may result from a defect in the activation of the intra-S phase checkpoint [34]. As such, we examined whether H2Bub interacts with factors that stabilize the replication fork during replication stress, by systematically examining the genetic interactions between *htb-K123R* and mutations in key components of this complex signaling system. Initially, we examined a hypomorphic allele of *pol2-11*, which encodes a mutant form of Polε that causes defects in the intra-S phase checkpoint [44]. The *htb-K123R* and *pol2-11* double mutant exhibited synthetic growth defects at the permissive temperature (23°C) (Fig. 4A, top left panel). This interaction was confirmed to be specific, because double mutants of *htb-K123R* and *pol1-17*, *pol3-14*, or *pri2-1* (replication defective mutants of DNA polymerase α and δ, and RNA primase, respectively [45]), exhibited subtle additive growth defects, or sensitivity to 50 mM HU at both the permissive (23°C; Fig. S4A) and non-permissive (30°C; Fig. S4B) temperature for growth.

We next examined the effect of HU on strains containing *htb-K123R* and *mec1-100*, an intra-S phase checkpoint defective allele of Mec1/ATR [46], or deletion of *MRC1* or *SGS1* (Fig. 4A). Single mutants of *htb-K123R* and *mec1-100* grew in the presence of 10 and 25 mM HU, but the double mutant was highly sensitive to these concentrations of HU (Fig. 4A, top right panel). Interestingly, similar phenotypes were observed upon combining *htb-K123R* with deletions of the genes encoding the checkpoint mediator protein Mrc1 or the RecQ helicase Sgs1 (Fig. 4A, bottom left panel), suggesting that H2Bub stabilizes the replication fork independently of these proteins. We then examined whether H2Bub interacts with the kinase checkpoint effector, Rad53. The *rad53-11* mutant is checkpoint defective, with undetectable Rad53 activity [47]. Intriguingly, the hypersensitivity of *rad53-11* to HU was partially reversed by *htb-K123R* (Fig. 4A, bottom-right panel). Deletion of the H2Bub-specific E3 ligase Bre1 had similar effects to *htb-K123R* when combined with the *mec1-100*, *sgs1Δ*, or *rad53-11* mutations (Fig. 4B, S4C), suggesting that the genetic interactions between H2Bub and components required for the re-initiation of stalled forks are at the level of chromatin structure, and are linked to its chromatin modifying activities. We next examined the effect of H2Bub deficiency on the viability of *mec1-100* and *rad53-11* cells in the presence of HU. The absence of H2Bub exacerbated the lethality observed in *mec1-100* cells in S phase, while enhancing the viability of *rad53-11* cells under the same conditions (Fig. 4C). Overall, our genetic analyses suggest that H2Bub and the Mec1-dependent S-phase checkpoint function in parallel to preserve fork stability under replication stress. However, our finding that *htb-K123R* alleviates the *rad53-11* growth defect under HU suggest that that H2Bub may function upstream of Rad53 and participate in the replication stress response.

### H2Bub and Sgs1 play interdependent roles in the replication stress response

**Figure 5:**
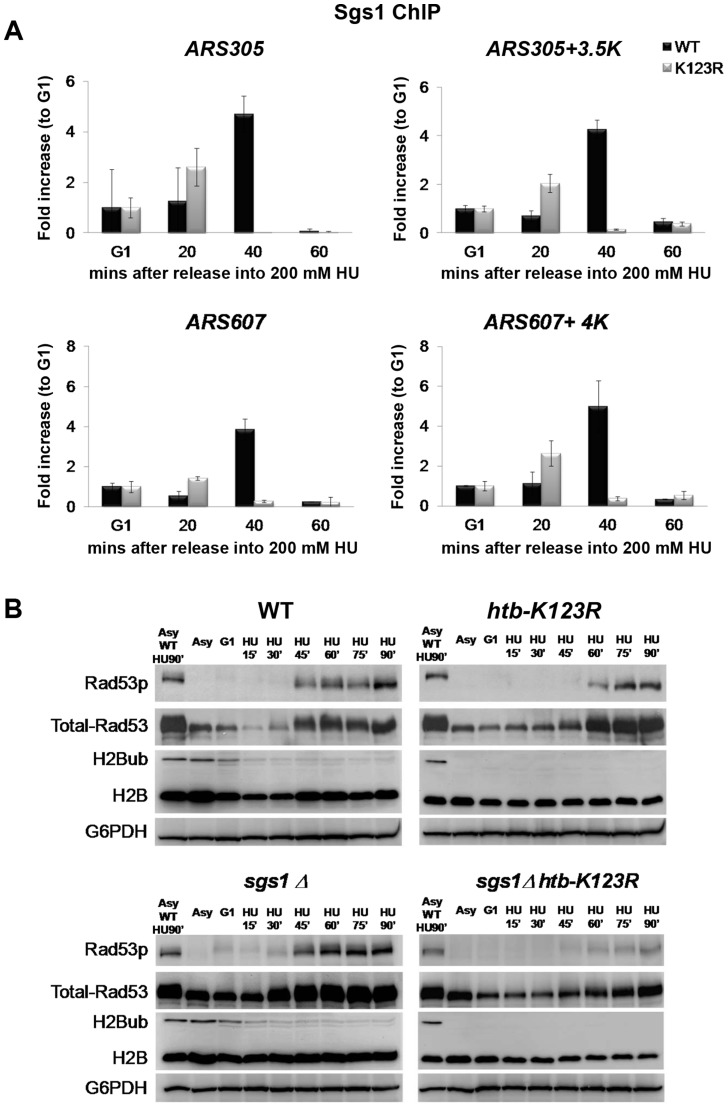
H2Bub and Sgs1 play interdependent roles in Rad53 phosphorylation. (A) Sgs1 occupancy at replication origins is unstable in *htb-K123R* cells exposed to HU. WT (CFK1764) or *htb-K123R* (CFK1765) cells were synchronized in G1 and then released into fresh YPD containing 0.2M HU for 60 minutes at 30°C. Chromatin immunoprecipitation (ChIP) was performed using antibodies against Sgs1-3×Myc. DNA was quantified by qPCR using primers adjacent to ARS305 and a region 3.5 kb distal. Sgs1 occupancy at each time point was normalized to that of G1. (B) Activation of Rad53 is impaired in the absence of both H2Bub and Sgs1. WT (CFK1204), *htb-K123R* (CFK1231), *sgs1Δ* (CFK1447), and *sgs1Δ htb-K123R* (CFK1453) cells were arrested in G1 and released into fresh media containing 0.2M HU for 90 minutes at 30°C. Whole cell lysates were prepared at the indicated time points, and analyzed by Western blot using antibodies against Rad53 (EL7), phospho-Rad53 (F9), H2B, and mono-ubiquitylated H2B (anti-FLAG). G6PDH was used as a loading control.

Comparing our data with earlier works [37], [48], [49] revealed several lines of evidence which suggest that H2Bub and the RecQ helicase Sgs1 have overlapping functions in maintaining fork stability under HU. First, both *htb-K123R* and *sgs1Δ* cells exhibit increased fork progression in HU (Fig. 1F; [37]). Second, the absence of either Sgs1 or H2Bub reduces the stability of stalled replication forks under HU (Fig. 1A and 3C; [48]). Third, the combination of *htb-K123R* or *sgs1Δ* with *mec1-100* causes fork collapse and failure to recover from acute exposure to HU (Fig. 4C; [49]). To better delineate the role of H2Bub in the replication stress response, we decided to investigate the interaction between H2Bub and the Sgs1 helicase further. We used ChIP to measure the recruitment of Sgs1 to the *ARS305* and *ARS607* early origins in WT and *htb-K123R* cells (Fig. 5A). While Sgs1 was initially recruited efficiently to *ARS305* and *ARS607* in both strains, it failed to accumulate at *ARS* in the mutant, suggesting the association of Sgs1 with the replication fork was unstable in the absence of H2Bub (Fig. 5A). Furthermore, HU-induced phosphorylation of Rad53 was unaffected in *sgs1Δ*, but delayed in *htb-K123R* cells (Fig 5B). Rad53 activation is facilitated by the retention of Sgs1 at stalled forks [33], and the current results suggest that H2Bub may be required for such retention, and thus Rad53 phosphorylation. Interestingly, a recent study demonstrated that RPA-coated single-stranded DNA replication intermediates (ssDNA) are reduced at initiated origins in *htb-K123R* cells under HU [28]. RPA is postulated to interact with Sgs1 at replication forks [50]. Thus, the reduced Sgs1 occupancy at replication forks and delayed Rad53 phosphorylation in the *htb-K123R* mutant may be explained by the decreased amount of ssDNA at replication forks. In addition, Rad53 phosphorylation was significantly impaired in a *sgs1Δ htb-K123R* double mutant (Fig. 5B). This is also indicative of a Sgs1-independent role for H2Bub in Rad53 activation. Collectively, these results point to a functional role for H2B in replication and the checkpoint response, and are consistent with the observed epistatic interaction between H2Bub and Rad53 (Fig. 4A–C).

### H2Bub and Sgs1 cooperatively control replication fork stalling under HU

**Figure 6:**
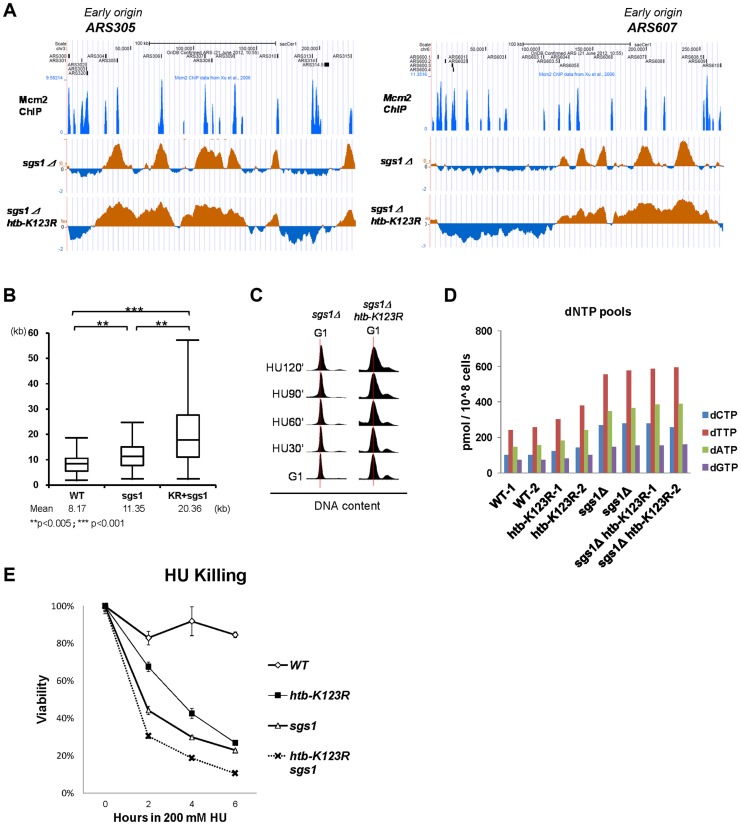
H2Bub and Sgs1 cooperatively control replication fork stalling and stability under HU. (A) Replication profiles of the early origins *ARS305* and *ARS607* in *sgs1Δ* (YCL007) and *sgs1Δ htb-K123R* (YCL008) mutants. The BrdU histogram was analyzed as described in Fig. 1C–E. (B) Graph depicting the distribution of BrdU track lengths in WT (CFK1419), *sgs1Δ* (YCL007), and *sgs1Δ htb-K123R* (YCL008) mutants, as shown in Fig. 1F. (C) Cell cycle progression of these mutants in the presence of 0.2M HU was analyzed by flow cytometry. (D) The size of the dNTP pools in exponentially-growing cultures of WT (CFK1419), *htb-K123R* (CFK1421), *sgs1Δ* (YCL007), and *sgs1Δ htb-K123R* (YCL008) cells in YPD media. Two independent isogenic strains of each genotype were analyzed. (E) Survival of WT (CFK1204), *htb-K123R* (CFK1231), *sgs1Δ* (CFK1447), and *sgs1Δ htb-K123R* (CFK1453) cells in response to acute doses of HU, as shown in Fig. 1A.

To further elucidate the interaction between Sgs1 and H2Bub, we used BrdU IP-chip to monitor fork progression in s*gs1Δ* and s*gs1Δ htb-K123R* mutant cells in the presence of HU. Consistent with a previous report [37], the average BrdU track length in *sgs1Δ* cells was significantly increased as compared to WT cells (11.35 kb vs. 8.17 kb, respectively; Fig. 6A and 6B), similar to the increase observed in *htb-K123R* cells (Fig. 1F). Remarkably, track lengths in the s*gs1Δ htb-K123R* double mutant (20.36 kb) were even greater, being almost 2.5-fold longer than those in WT (8.17 kb; Fig. 6A and 6B). Flow cytometry was used to confirm that the double mutant contained greater amounts of DNA in the presence of 200 mM HU (Fig. 6C). Increased fork progression in the absence of Sgs1 is believed to be a consequence of dNTP accumulation [37]. We confirmed that the dNTP concentration was increased in s*gs1Δ* (∼2.3 fold as compared to WT; Fig. 6D), but no additional increase was observed in the *sgs1Δ htb-K123R* double mutant, suggesting that elevated dNTP production does not underlie the defect in fork stalling in the double mutant.

We have demonstrated that the replication fork becomes unstable and vulnerable to replication stress in H2Bub-deficient cells (Fig. 1A & 3B), which could be due to continuous DNA synthesis under conditions of dNTP depletion. Thus, we reasoned that the rapidly moving replication fork may become highly unstable in the absence of both H2Bub and Sgs1, a hypothesis supported by the observation that the double mutant was more sensitive to acute treatment with HU than either single mutant (Fig. 6E). Taken together, our results so far suggest that H2Bub is involved in stalling the replication fork and maintaining its stability in response to HU-induced S phase block; furthermore, this function is performed in cooperation with Rad53 kinase activity and in parallel with Mec1 and Sgs1 during S phase.

### H2Bub promotes chromatin assembly in response to replication stress

**Figure 7:**
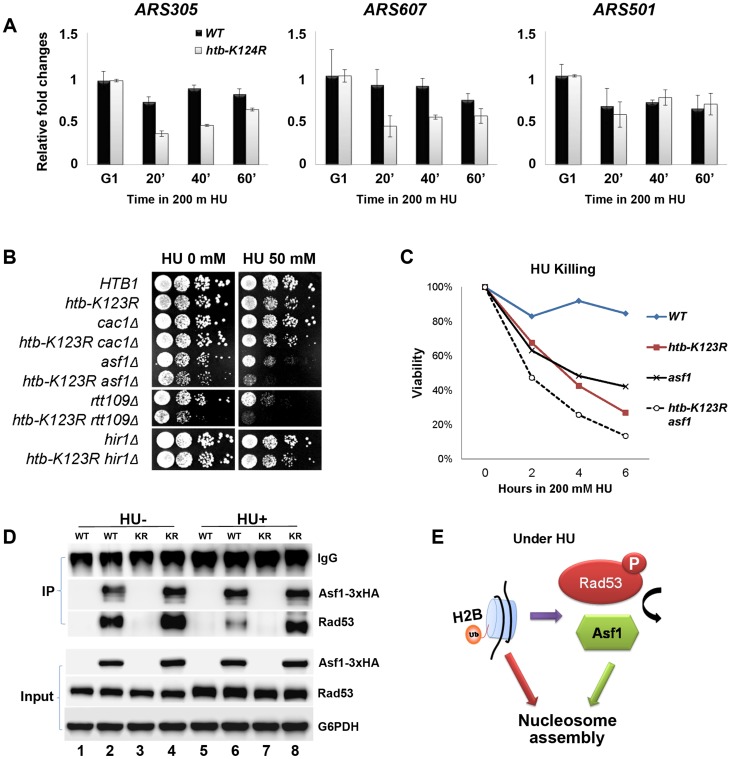
H2Bub promotes chromatin assembly in response to replication stress. (A) H2Bub is required for nucleosome assembly near replication forks under replication stress. WT (CFK1204) or *htb-K123R* (CFK1231) cells were arrested in G1 phase using α-factor, and were then released into 200 mM HU at 30°C for 60 minutes. At the indicated time, cells were collected and histone occupancy at two early origins (*ARS305* and *ARS607*) and one late origin (*ARS501*) was determined by ChIP using antibodies against H3. IP signals at ARS sequences were normalized to IP signals at TELVI-R. The results are the mean +/− SEM of three replicates. (B) Genetic interactions between H2Bub and histone chaperones (Cac1, Asf1, and Hir1) or a histone acetyl-transferase (Rtt109). Ten-fold serial dilutions of the indicated strains (WT (CFK1204), *htb-K123R* (CFK1231), *cac1Δ* (CFK1206), *cac1Δ htb-K123R* (CFK1237), *asf1Δ* (CFK1208), *asf1Δ htb-K123R* (CFK1233), *rtt109Δ* (CFK1212), *rtt109Δ htb-K123R* (CFK1241), *hir1Δ* (CFK1202), and *hir1Δ htb-K123R* (CFK1235)) were spotted onto YPD plates containing HU (0 or 50 mM), and cell growth was monitored for 2–3 days. (C) The survival of asf1Δ (CFK1208) and *asf1Δ htb-K123R* (CFK1233) cells in response to acute treatment with HU, as described in Fig. 1A. (D) H2Bub modulates the interaction between Asf1 and Rad53 under HU stress. Asynchronous cultures of WT (YMW105) or *htb-K123R* (YMW104) cells were untreated (−) or treated (+) with 0.2M HU for 90 minutes. Protein extracts were prepared and incubated with pre-bound anti-HA-protein G beads to pull down Asf1-3×HA, and the immune-precipitates were resolved by SDS-PAGE, before being probed with either anti-HA or anti-Rad53 antibodies. (E) A working model depicting the role of H2Bub in nucleosome assembly under HU stress. H2Bub coordinates nucleosome assembly in response to replication stress by directly contributing to nucleosome formation and by indirectly regulating the availability of Asf1 during HU stress.

H2Bub has been shown to be required for nucleosome reassembly during RNA Polymerase II elongation [20], [23] and DNA replication [28]. We therefore hypothesized that defective fork stalling in *htb-K123R* cells under replication stress may be a consequence of incomplete nucleosome assembly. We first confirmed that histone assembly on newly-synthesized DNA is defective in *htb-K123R* under HU. In WT cells, histone H3 was associated with the early firing origins *ARS305* and *ARS607* at all times post-G1 release into HU. H3 occupancy at these early origins was reduced upon entry into S phase in *htb-K123R* cells, but occupancy at the late origin *ARS501* was unaffected (Fig. 7A). These data suggest that in the absence of H2Bub, histone assembly is less efficient at firing origins.

Mec1 was recently reported to increase chromatin accessibility at or ahead of replication forks, and promote fork progression in HU [35]. Thus, the mechanism promoting nucleosome assembly during DNA replication may inhibit fork progression under replication stress. We reasoned that if this were the case, deletion of genes encoding proteins involved in replication-coupled histone assembly (such as the histone chaperones CAF-1 and Asf1) should sensitize *htb-K123R* cells to replication stress. Asf1 has dual roles; it associates with the RCF complex and MCM helicase and facilitates nucleosome disassembly during replication [51], [52], and it assists acetylation of H3 lysine 56 (H3K56ac) by presenting newly-synthesized H3/H4 dimers to the Rtt109 acetyltransferase [4], [53]. Acetylation increases the affinity of H3 for CAF-1 [53] and promotes efficient chromatin assembly onto nascent DNA [8]. As a control, we deleted Hir1; this protein is implicated in replication-independent H3/H4 deposition [54]. We found that deletion of *ASF1* or *RTT109* greatly increased the HU sensitivity of *htb-K123R* cells, but deletion of *CAC1* (the largest subunit of CAF-1) or *HIR1* had no such effect (Fig. 7B). This suggests that H2Bub and Asf1-Rtt109 function synergistically to promote cell survival during replication stress. Moreover, deletion of Asf1 increased the sensitivity of *htb-K123R* cells to acute HU treatment (Fig. 7C), suggesting that the stability of the replication fork was decreased further.

### H2Bub-mediated Rad53 activation promotes the dissociation of histone chaperone Asf1 from the Rad53 complex

Rad53 and Asf1 form a dynamic complex that dissociates in response to Rad53 phosphorylation under replication stress. Rad53 acts as a regulator of Asf1 availability and indirectly controls its chromatin assembly activity [55], [56]. Our results suggest that H2Bub may affect Rad53 phosphorylation (Fig. 5B). We hypothesized that H2Bub may contribute to nucleosome assembly by influencing the dynamic association between Asf1 and Rad53, in addition to possessing a direct role in nucleosome assembly. To test this hypothesis, we tagged the genomic *ASF1* gene with a triple HA tag, thereby enabling immunoprecipitation of Asf1 with an anti-HA antibody (Fig. 7D, lanes 2, 4, 6, and 8). Rad53 co-precipitated with HA-tagged Asf1 efficiently in WT lysates (Fig. 7D, lane 2) but not with un-tagged Asf1 (Fig. 7D, lanes 1, 3, 5, and 7). Consistent with previously published results [55], [56], Rad53 association with Asf1 was reduced in the presence of HU in WT cells (Fig. 7D, lane 6). However, the association of Rad53 with Asf1 remained stable in *htb-K123R* cells in the presence of HU (Fig. 7D, compare lanes 6 and 8). These results suggest that H2Bub coordinates nucleosome assembly in response to replication stress by directly contributing to nucleosome formation, and by indirectly regulating the availability of Asf1, which in turn deposits histones behind the advancing replication fork (Fig. 7E).

## Discussion

Here, we report that replication fork stalling is regulated by the Bre1-H2Bub pathway in the presence of HU-induced stress. We demonstrate that elimination of H2Bub enhances replication fork progression and instability in HU. Importantly, this process is independent of Dun1-mediated regulation of dNTP pools. Instead, H2Bub promotes Rad53 activation and mediates dissociation of phosphorylated Rad53 and Asf1, which may contribute to nucleosome assembly and promote cell survival in HU. These findings lead us to suggest that H2Bub plays a more direct role in fork stalling and stability under replication stress.

### Chromatin state facilitates tight regulation of fork progression during replication stress

How does the Bre1-H2Bub pathway modulate the cellular response to HU-induced replication block? Interestingly, H2B ubiquitylation has been proposed to promote unwinding of the DNA chromatin complex ahead of the replication fork, and thereby stimulate fork progression in HU [28]. However, our results support an alternative role for H2Bub in restricting replication fork progression under conditions of HU stress.

**Figure 8:**
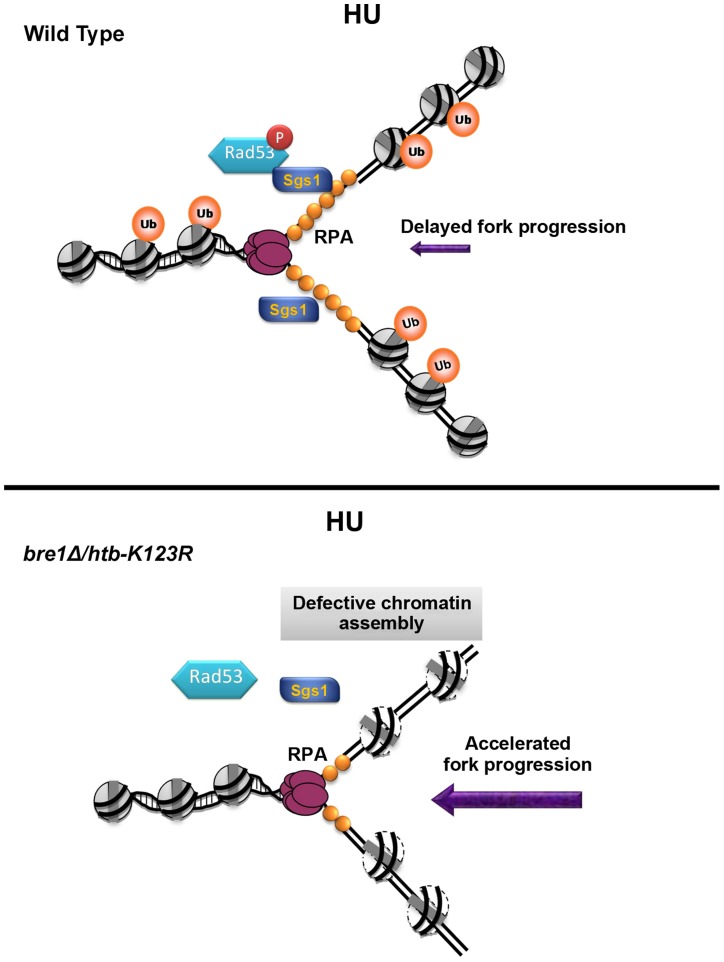
A model for how H2B mono-ubiquitylation facilitates fork stability under replication stress. Upon HU-induced stress, H2Bub promotes nucleosome assembly, which assists replication fork stalling, Sgs1 recruitment, and Rad53 phosphorylation. The reassembly of chromatin on nascent DNA restricts fork progression and promotes replication fork stability and its recovery after the removal of HU. In the absence of H2Bub (*bre1Δ/htb-K123R*), replication fork movement is faster than even that observed under nucleotide depletion by HU, which results in shorter tracts of RPA-coated-single-stranded DNA. This in turn reduces retention of Sgs1 at the forks, and delays phosphorylation of Rad53.

We found that histone occupancy around early origins in *htb-K123R* cells is reduced upon S phase entry in the presence of HU (Fig. 7A). In addition, we also showed that the removal of both Asf1-Rtt109 and H2Bub synthetically increases the sensitivity to replication stress (Fig. 7B and C). Moreover, we provide evidence that H2Bub controls the availability of Asf1 during replication stress (Fig. 7D), which potentially contributes to histone deposition behind the advancing replication fork [55], [56]. Thus, we propose that enhanced chromatin assembly on nascent DNA during replication stress may facilitate replication fork stalling in response to nucleotide depletion imposed by HU (Fig. 8), akin to the brakes on a train (the replisome). Mec1 slows S phase progression by delaying late origin firing through intra-S phase checkpoint activation [30], [34], but it also promotes sustained replication fork progression at early origins [35]. The chromatin state seems to facilitate tight regulation of fork progression at early origins during replication stress.

We cannot exclude the possibility that continuous DNA synthesis in the *htb-K123R* mutant may reflect the movement of DNA polymerase through inappropriately-assembled chromatin. In this scenario, chromatin structure at or ahead of the fork would be altered in the absence of H2Bub due to defective chromatin assembly during the previous round of replication. Mec1 and Bre1-H2Bub may have antagonistic effects on chromatin dynamics ahead of the replication fork; thus in the absence of H2Bub, forks may be inclined to move faster because of Mec1-induced chromatin accessibility [35]. Although the scenario outlined above is formally possible, our molecular and genetic analyses favor a second model in which nucleosome formation on nascent DNA serves as a negative feedback mechanism to regulate the progression of the replication fork under stress. Thus, we suggest that Mec1-mediated signaling and the Bre1-H2Bub pathway synergistically interact to ensure that replisomes travel in a controlled manner, thereby maintaining fork stability under replication stress.

### H2Bub is a regulator of the DNA replication stress signaling pathway

Checkpoint kinases Mec1 and Rad53 are essential for the maintenance of cell viability when replication is perturbed [30], [31]. Our genetic analyses reveal the unexpected finding that H2Bub maintains fork stability in parallel with the Mec1-mediated intra-S checkpoint, but its effect is epistatic to that of a second checkpoint kinase, Rad53. Our results support the hypothesis that Rad53 stabilizes replication forks independently of Mec1 [57], [58]. Furthermore, our findings suggest a possible mechanism for the role of H2Bub in Rad53 activation (Fig. 8 upper panel). We report that the stable association of Sgs1 with the replication fork is not only replication-dependent [48], but also H2Bub-dependent (Fig. 5A). It was previously demonstrated that Sgs1 helps recruit Rad53 to stalled forks via an interaction with RPA [50]. Intriguingly, it has been postulated that fork collapse followed by origin firing in yeast cells lacking H2Bub results in reduced levels of single-stranded DNA (ssDNA) and RPA during a G1 to HU shift [28], consistent with our observation of reduced replication intermediates and increased DNA damage in *htb-K123R* cells (Fig. 3). However, it is also possible that the failure to accumulate RPA in *htb-K123R* cells may be caused by an increase in the rate of nascent DNA synthesis, thereby reducing the accumulation of ssDNA at stalled forks; this in turn reduces Sgs1 retention and delays Rad53 phosphorylation (Fig. 8, bottom panel). The reduced activity of Rad53 may have a negative feedback effect, thereby further compromising fork stability. The absence of both H2Bub and Sgs1 therefore further disrupts Rad53 activation and fork integrity.

### The mechanism by which chromatin assembly regulates fork progression and stability

In support of our model that chromatin assembly serves as a negative feedback signal to regulate the progression of replication forks, several reports in budding yeast have established that chromatin assembly at replication forks is necessary to stabilize replication forks and prevent their collapse [52], [59], [60]. A recent report in mammals demonstrated that replication fork speed is dependent on the supply of new histones and efficient nucleosome assembly during an unperturbed cell cycle [61]. Human Asf1 has been shown to associate with the MCM complex through histone H3/H4 dimers [51]. In addition, Asf1 extracted from human cells exposed to HU exhibits an enhanced ability to assemble chromatin [62]. Thus, there may be two pools of Asf1 in cells. One is coupled to replication forks, while the other is sequestered by Rad53. Replication stress triggers the release of the sequestered pool of Asf1 (which occurs at least in part through H2Bub) to promote chromatin formation (Fig. 7E) and restrict fork progression (Fig. 1C–F) under replication stress. However, defects in nucleosome assembly mediated by CAF-1 trigger DNA damage checkpoint activation and delay fork progression in human cells during an unperturbed cell cycle [63]–[65]. Our genetic analysis shows that Cac1, unlike H2Bub and Asf1, is not required by yeast cells to maintain growth under HU stress; hence chromatin assembly regulated by H2Bub and Asf1 under replication stress (Fig. 7B) likely occurs through pathways distinct from those mediated by CAF-1 [66].

In summary, we have provided evidence that H2Bub coordinates chromatin assembly and Rad53 activation during HU stress in parallel with other mechanisms that maintain fork stalling and stability during replication stress, including the intra-S phase checkpoint and the Sgs1 helicase. Our data indicate that H2Bub maintains genomic stability by creating an environment that integrates chromatin formation and checkpoint kinase activation, thereby maintaining stable replication and facilitating recovery from replication stress in concert with other components that mediate faithful DNA replication.

## Materials and Methods

### Yeast strains, plasmids, and phenotypic screening

Yeast strains and plasmids used in this study are shown in supplementary tables S1 and S2. All yeast cells were cultured in yeast extract peptone supplemented with 2% dextrose at 30°C. All analyses were performed during the log phase of growth. Cells were arrested in G1 by the addition of α-factor to a final concentration of 100 ng/ml (*bar1Δ* strain) for at least 3 hours (the exact time differed depending on the strain). Cells were released from G1 arrest by washing with sterilized H_2_O three times, before being re-suspended in fresh media containing hydroxyurea (HU; Sigma).

For phenotypic screening, mid-log (0.4–0.8) phase cultures were collected and counted. Ten-fold serial dilutions were spotted onto YPD plates containing different doses of HU. Plates were subsequently incubated at 30°C for several days.

Two different strain backgrounds were used in this study. With the exception of the strains used in the genetic analysis shown in Fig. 4, all strains were in the YS131 background. The YS131 parental strain is derived from W303, but both genomic copies of HTA1-HTB1 and HTA2-HTB2 are deleted, and cell viability is maintained by a plasmid-derived HTA1-HTB1 or HTA1-htb1-K123R. Earlier studies established that deletion of HTA2-HTB2 has negligible effects on mitotic growth and stress responses, and that the HTA1-HTB1 gene pair can compensate for the absence of the HTA2-HTB2 [67], [68]. Therefore, we predict that hta2-htb2Δ would not affect the *htb-K123R* mutation.

For the genetic analysis with the checkpoint mutants, we were conscious of an earlier report that the rad53 mutant is sensitive to histone dosage [69]. To prevent unexpected pleiotropic effects, we introduced genomic *htb-K123R* mutations [*HTA1-htb1-K123R::NAT+ HTA2-htb2-K123R::HIS+*] [70] into mec1-100 and rad53-11 for genetic analysis. We also compared the HU sensitivity of the *htb-K123R* mutants in both strain backgrounds to ensure that they give rise to the same replication defects, as shown in Fig. S5.

### Gene replacement

For gene disruptions, the indicated gene was deleted by high efficiency transformation, using a PCR product in which the target was replaced with the *KanMX* gene (deletion library from SGD). The mutant alleles, pol1-17 [45], pri2-1 [45], pol2-11 [71] and pol3-14 [45], were introduced into strain CFK1204 or CFK1231 through the gene replacement technique of Scherer and Davis [72], thereby generating *ts* mutants. The plasmid used for gene replacement consisted of a 9-kb *pol1*(Ts), 3.3-kb *pri2*(Ts), 13-kb *pol2*(Ts), or 4-kb *pol3*(Ts) fragment cloned into the *Xho*I site of YlP, *Hpa*I site of YlPA16, *Age*I site of pRS306, or *Kpn*I site of pMJ14.

### BrdU-IP chip analysis

*S. cerevisiae* strains were designed in order to allow BrdU incorporation (TK repeats) (Tables S1 and S2). *S. cerevisiae* oligonucleotide microarrays were obtained from Affymetrix. BrdU-IP chip analysis was carried out as previously described [73], [74]. Briefly, cells were synchronized with α-factor and then released into fresh YPD containing 0.2M HU and 200 µg/ml BrdU for 90 minutes. The collected cells were arrested in ice-cold buffer containing 0.1% Na-azide, and genomic DNA was extracted from 2×10^9^ cells as described in the “QIAGEN Genomic DNA Handbook”. DNA was sheared to 300 bp by sonication, denatured, and mixed with 4 µg anti-BrdU monoclonal antibody (MBL M1-11-3) as previously described [75], [76]. Antibody-bound and unbound fractions were subsequently purified, and then amplified using the WGA2 GenomePlex Complete Genome Amplification Kit. A total of 2 µg of amplified DNA was digested with DNaseI to a mean size of 100 bp; the fragments were subsequently end-labeled with biotin-N11-ddATP [77], and hybridized to the DNA chip.

### Flow cytometry analysis

For DNA content analysis, approximately 1×10^7^ cells were collected at each time point, and resuspended in 1 ml 70% ethanol (ice-cold), before being stored at −80°C for at least one night (samples were stored up to a maximum of 3 days). The cells were then washed twice with 1 ml 50 mM Tris-HCl (pH 8.0) followed by RNAase A digestion (1 mg ml^−1^ of RNAase A in 50 mM Tris-Cl, pH 8.0) and proteinase K digestion (16 units ml^−1^ in 30 mM Tris-Cl, pH 8.0). Finally, cells were stained with SYBR GREEN I buffer (in 50 mM Tris-Cl, pH 8.0) at 4°C overnight. The cell size and DNA contents of 50,000 cells were examined on a FACSCanto II (BD).

### Two-dimensional (2D) electrophoresis and Southern blot

Total genomic DNA was extracted according to the protocol of the QIAGEN Genomic DNA Handbook, using genomic-tip 100/G columns. 2D gel electrophoresis was carried out as originally described by Brewer and Fangman [78]. The DNA samples were digested with HindIII or SacI/ApaL1, for *ARS305* and *ARS607* detection respectively, and then blotted onto a Nylon Gene Screen Plus membrane (NEN). Membranes were probed with the *Bam*HI-*Nco*I 3.0 kb fragment which spans *ARS305* and was purified from plasmid A6C-110 (kindly provided by C. Newlon, uMDNJ, Newark, NJ), or probed with a 3.0 kb PCR product that spans *ARS607*. Signals were detected using a PhosphorImager Typhoon FLA 7000 (GE Healthcare).

### HU survival assay

To determine viability in response to acute doses of HU, cells were grown in culture media until they reached log phase. The cells were then arrested in G1 for 3 hours by the addition of α-factor, before being released into rich media containing 200 mM HU. Aliquots were removed from each culture at the indicated time point, plated onto YPD plates, and allowed to grow at 30°C for 2–3 days. Viability was estimated based on colony forming unit (CFU) counts, and was adjusted to that of wild-type at each time point.

### Western blot

Yeast cell lysates were prepared using the TCA method [79]. Briefly, equivalent numbers of cells (1.5×10^8^) were collected, resuspended in 200 µl TCA buffer (1.85 M NaOH and 7.4% β-mercaptoethanol), and placed on ice for 10 minutes. Following the addition of 200 µl of 20% TCA, the lysates were incubated on ice for 10 minutes. Pellets were subsequently collected, washed with 1 ml acetone, dried, and dissolved in 200 µl 0.1 M NaOH. The concentration of each sample was determined, and equal amounts were separated by SDS-PAGE, before being transferred to PVDF membranes for immunoblotting. The following antibodies were used: anti-GAPDH (Sigma), anti-Flag (Sigma) and anti-phospho-Rad53 (produced and characterized by A. Pellicioli and the IFOM antibody facility, and kindly provided by Dr. Foiani [80]). Secondary antibodies conjugated to horseradish peroxidase were detected using enhanced chemiluminescence (Amersham Biosciences).

### Determination of dNTP pools

The dNTP pools were analyzed as described by a recent study [81]. At a density from 0.4 to 0.8×10^7^ cells/ml, ∼3.7×10^8^ cells were collected onto a 0.8 µm nitrocellulose filter (Millipore AB, Solna, Sweden). The filters were immersed in 700 ml of ice-cold extraction solution (12% w/v trichloroacetic acid, 15 mM MgCl_2_) in Eppendorf tubes. The following steps were carried out at 4°C. The tubes were vortexed for 30 s, incubated for 15 min, and vortexed again for 30 s. The filters were removed, and the solutions were centrifuged at 20,000× g for 1 min. After centrifugation, 700 ml of supernatant was added to 800 ml of ice-cold Freon–trioctylamine mixture [10 ml of 99% Freon (1,1,2-trichlorotrifluoroethane; Aldrich, Sigma-Aldrich Sweden AB, Stockholm, Sweden)], and 2.8 ml of>99% trioctylamine (Fluka, Sigma-Aldrich Sweden AB, Stockholm, Sweden). The samples were vortexed and centrifuged for 1 min at 20,000× g. The aqueous phase was collected and added to 700 ml of an ice-cold Freon–trioctylamine mixture. Aliquots (475 and 47.5 ml) of the resulting aqueous phase were collected. The 475 ml aliquots were pH adjusted with 1M NH_4_HCO_3_ (pH 8.9), loaded onto boronate columns [Affi-Gel 601 (Bio-Rad)], and eluted with 50 mM NH_4_HCO_3_, pH 8.9, 15 mM MgCl_2_ to separate dNTPs and NTPs. The eluates with purified dNTPs were adjusted to pH 3.4 with 6M HCl, and separated on a Partisphere SAX-5 HPLC column (125 mm×4.6 mm, 5 µm, Hichrom, UK) using the Hitachi HPLC EZChrom system. Nucleotides were isocratically eluted using 0.36M ammonium phosphate buffer (pH 3.4, 2.5% v/v acetonitrile). The 47.5 ml aliquots were adjusted to pH 3.4 and used to quantify NTPs by HPLC in the same way as dNTPs. The nucleotides were quantified by measuring the peak heights and comparing them to a standard curve.

### Chromatin immunoprecipitation (ChIP)

Yeast strains were grown to an OD_600_ of 0.4–0.8, and fixed with 1% formaldehyde at room temperature (RT) for 20 min. Fixation was stopped by the addition of glycine to a final concentration of 125 mM for 5 min, and the cells were then collected and washed twice with ice-cold TBS (100 mM Tris at pH 7.5, 0.9% NaCl). Cell pellets were stored at −80°C or resuspended immediately in 500 µl of FA lysis buffer (50 mM HEPES, pH 7.5, 140 mM NaCl, 1 mM EDTA, 1% sodium deoxycholate, 0.1% SDS) supplemented with fresh protease inhibitor cocktail (Sigma), and lysed by vortexing with glass beads for 30 min at 4°C. Cell lysates were sonicated in a cooling water bath four times for 10 min each using a SONICATOR 3000 (MISONIX), with each cycle consisting of 30 sec sonication on and 30 sec off. The average size of the resulting DNA fragments was between 200 and 500 base pairs. Following centrifugation at 13.5K for 30 min at 4°C, the solubilized chromatin was collected and adjusted to 500 µl with FA lysis buffer. Twenty microliters were removed for use as input chromatin.

For immunoprecipitation, 10 OD equivalents of solubilized chromatin were incubated overnight at 4°C, together with 20 µl of protein G dynabeads (Invitrogen) that had been pre-bound with anti-H3 or anti-Myc (Sgs1-13Myc). Immunoprecipitates were collected by a step-wise washing protocol, consisting of 1.5 ml FA-lysis buffer, 1.5 ml WASH I (FA lysis buffer+0.5 M NaCl), 1.5 ml WASH II (10 mM Tris-Cl, pH 7.5, 1 mM EDTA, 0.25 M LiCl, 0.5% NP-40, 0.5% sodium deoxycholate), and 1.5 ml TE (pH 8.0) for 5 min each at room temperature. The immuno-complexes were eluted by adding 0.25 ml Elution buffer (50 mM Tris-Cl, pH 7.5, 10 mM EDTA, 1% SDS), and incubated first at 65°C for 20 minutes, and then at room temperature for 10 minutes with vortexing. DNA was purified using Qiaquick PCR purification spin-columns (Qiagen), and used as template for quantitative-PCR. All the primers used is listed in Table S3. The primers used in the histone H3 ChIP experiment were designed to amplify DNA fragments present at nucleosomes, as depicted in Figure S6.

### Co-immunoprecipitation (Co-IP)

For immunoprecipitations [56], log phase WT or *htb-K123R* cells untreated (−) or treated (+) with 0.2M HU were collected, resuspended in buffer containing 50 mM Tris7.5, 150 mM NaCl, 5 mM EDTA, 0.5% Triton X-100, and proteinase inhibitors, and broken open by bead beating. A total of 5 mg of protein extract was diluted in 1 ml of the same buffer, and incubated with pre-bound anti-HA-protein G beads at 4°C for 2.5 hours, and then rotated at 4°C overnight. Beads were then washed with 1 ml buffer four times. SDS-loading dye was added, and samples were boiled and resolved on SDS-PAGE.

### Statistical analysis

Results are expressed as the mean ± SEM from the number of experiments indicated in the figure legends. Student's *t*-test was used to analyze statistical significance.

## Supporting Information

Figure S1 *(A–D)* Replication profiles in WT (CFK1419) vs. *htb-K123R* (CFK1421) cells. Cells were synchronized in G1 with α-factor, and then released into media containing 0.2M HU and 200 µg/ml BrdU for 90 minutes. After DNA extraction and fragmentation, BrdU-labeled DNA was immunoprecipitated and hybridized on high-resolution tiling arrays. Orange (*BrdU-IP*) histogram bars on the y axis show the average signal ratio on a log2 scale of loci along the reported regions on *(A)* chromosome II, *(B)* chromosome V, *(C)* chromosome IX, and *(D)* chromosome XVI. The positions of potential *ARS* elements are identified by Mcm2 loading.
(TIFF)

Figure S2 The size of each dNTP pool in exponentially-growing WT (CFK1419) and *htb-K123R* (CFK1421) cells. Four independent isogenic strains of each genotype were analyzed as described in the Materials and Methods.
(TIFF)

Figure S3 H2Bub is required for efficient origin firing. *htb1-K123R* mutants exhibit reduced BrdU incorporation during S phase. Cells were arrested at G1 using α-factor at 23°C, and released synchronously into S phase at 20°C in YPD supplemented with BrdU. Samples were collected at the indicated times and genomic DNA was then extracted. Monoclonal BrdU antibody was used to immunoprecipitate BrdU-incorporated DNA. DNA synthesis at replication origins (ARS305 and ARS607) or telomere was detected by quantitative-PCR. Cell cycle progression was monitored by FACS at 20°C under BrdU incorporation conditions.
(TIFF)

Figure S4 *(A)* The growth of *htb-K123R* and DNA polymerase *ts* double mutants are not affected by HU at the permissive temperature (23°C). Ten-fold serial dilutions of the indicated strains (WT (CFK1204), *htb-K123R* (CFK1231), *pol1-17* (CFK1984), *pol1-17 htb-K123R* (CFK1986), *pri2-1* (CFK1988), *pri2-1 htb-K123R* (CFK1990), *pol3-14* (CFK1992) and *pol3-14 htb-K123R* (CFK1994)) were spotted onto YPD containing different doses of HU (0–50 mM) at 23°C for several days. Growth at the restrictive temperature (37°C) is presented as a control for *ts* mutants. *(B)* The genetic interaction between H2Bub and DNA pol1, pol3, or primase. Ten-fold serial dilutions of the indicated strains were spotted onto YPD, and growth was monitored at 33°C or 30°C, or under conditions of replication stress (50 mM HU) at 30°C. *(C)* The histone H2B ubiquitin E3 ligase Bre1 functions in parallel with the RecQ helicase Sgs1 under replication stress. Ten-fold serial dilutions of the indicated strains (WT (CFK1204), *bre1Δ* (CFK1443), *sgs1Δ* (CFK2371) and *bre1Δ sgs1Δ* (CFK2373)) were spotted onto YPD containing different doses of HU (0–100 mM) at 30°C.
(TIFF)

Figure S5 The growth of WT and *htb-K123R* cells of two different backgrounds under conditions of replication stress at 30°C. Ten-fold serial dilutions of the indicated strains (WT (CFK1204), *htb-K123R* (CFK1231), WT (CFK2414), and *htb-K123R* (CFK2416)) were spotted onto YPD containing different doses of HU (0–150 mM) for 2 days. Genotypes of the strains used: CFK1024: W303 *hta1-htb1Δ hta2-htb2Δ <pZS145-HTA1-Flag-HTB1 CEN HIS3>* CFK1031: W303 *hta1-htb1Δ hta2-htb2Δ <pZS146-HTA1-Flag-htb1-K123R CEN HIS3>* CFK2414: W303 CFK2416: W303 *HTA1-htb1-K123R::NAT+ HTA2-htb2-K123R::HIS+*.
(TIFF)

Figure S6 **(A)** A schematic description of the nucleosome position surrounding *ARS305*, and the primers used in Fig. 7A to amplify *ARS305* for histone chromatin immunoprecipitation. *ARS305* (nuc.) (39,349–39,455) primer sequence: (F): att tca gag cct tct ttg gag, (R): atg aaa ctg gac ata ttt gag gaa. (**B**) A schematic description of the nucleosome position surrounding *ARS607*, and the primers used in Fig. 7A to amplify *ARS607* for histone chromatin immunoprecipitation. *ARS607* (nuc.) (199,539–199,630) primer sequence: (F): aca cat tat tcg gca cag tag, (R): tcg cag tcc ata gaa gga g. (**C**) A schematic description of the nucleosome position surrounding *ARS501*, and the primers used in Fig. 7A to amplify *ARS501* for histone chromatin immunoprecipitation. *ARS501* (nuc.) (549,785–549,858) primer sequence: (F): ctcct catca tcatc cc, (R): cgtac actag cccgt tg. Image created using the following software available at the Penn State Genome Cartography Project http://atlas.bx.psu.edu/cj/nucl_retrieval.html [82]
(TIFF)

Table S1 Yeast strains used in this study.
(PDF)

Table S2 Plasmids used in this study.
(PDF)

Table S3 Primers used in this study.
(PDF)
