# Supplementary figures and images for: H2B Mono-ubiquitylation Facilitates Fork Stalling and Recovery during Replication Stress by Coordinating Rad53 Activation and Chromatin Assembly
Source: PLoS Genet. 2014 Oct 2;10(10):e1004667. doi: 10.1371/journal.pgen.1004667 (PMC4183429; doi:10.1371/journal.pgen.1004667)

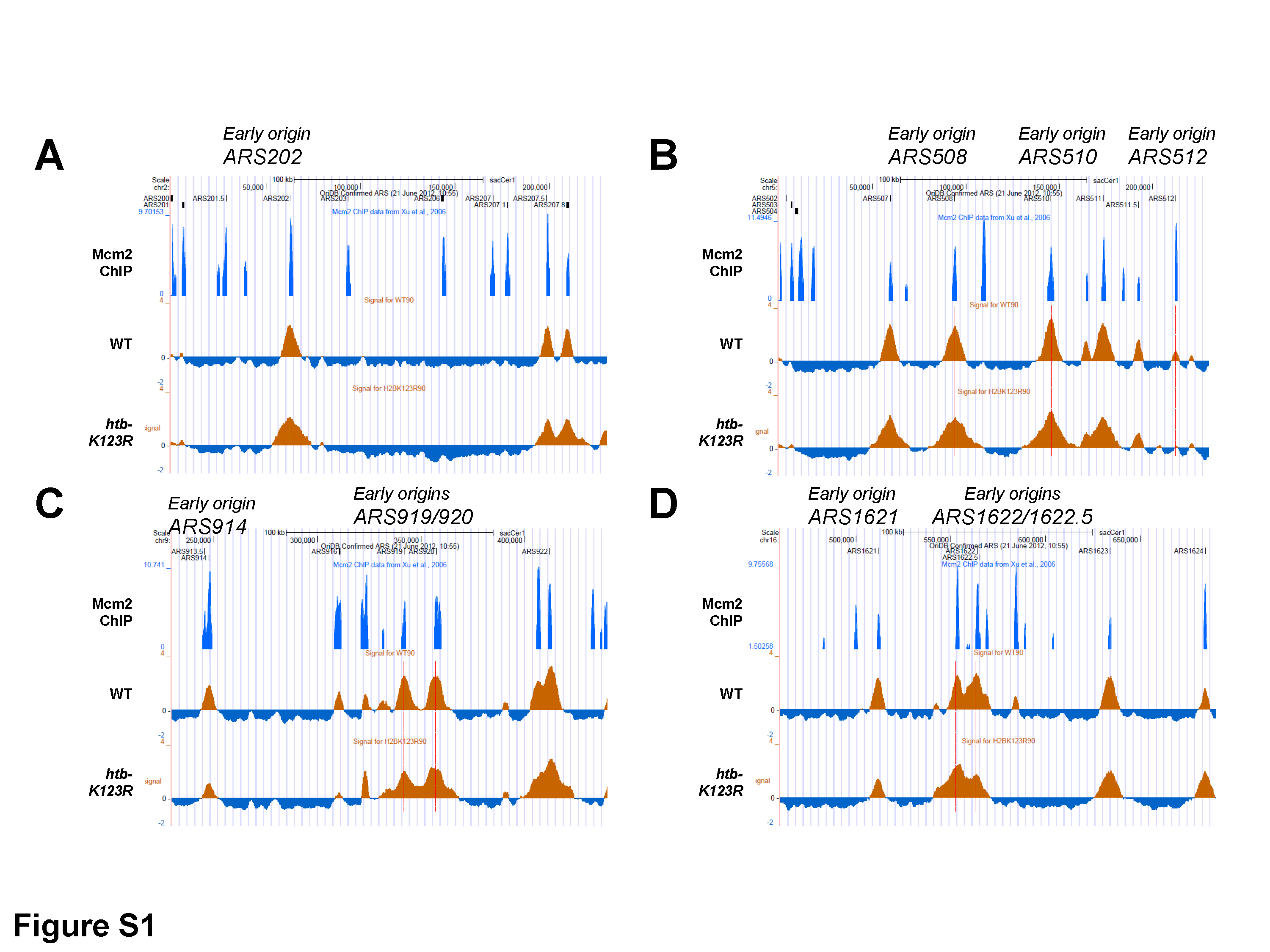

Supplement: Figure S1 — (A–D) Replication profiles in WT (CFK1419) vs. htb-K123R (CFK1421) cells. Cells were synchronized in G1 with α-factor, and then released into media containing 0.2M HU and 200 µg/ml BrdU for 90 minutes. After DNA extraction and fragmentation, BrdU-labeled DNA was immunoprecipitated and hybridized on high-resolution tiling arrays. Orange (BrdU-IP) histogram bars on the y axis show the average signal ratio on a log2 scale of loci along the reported regions on (A) chromosome II, (B) chromosome V, (C) chromosome IX, and (D) chromosome XVI. The positions of potential ARS elements are identified by Mcm2 loading. (TIFF) [file pgen.1004667.s001.tiff]

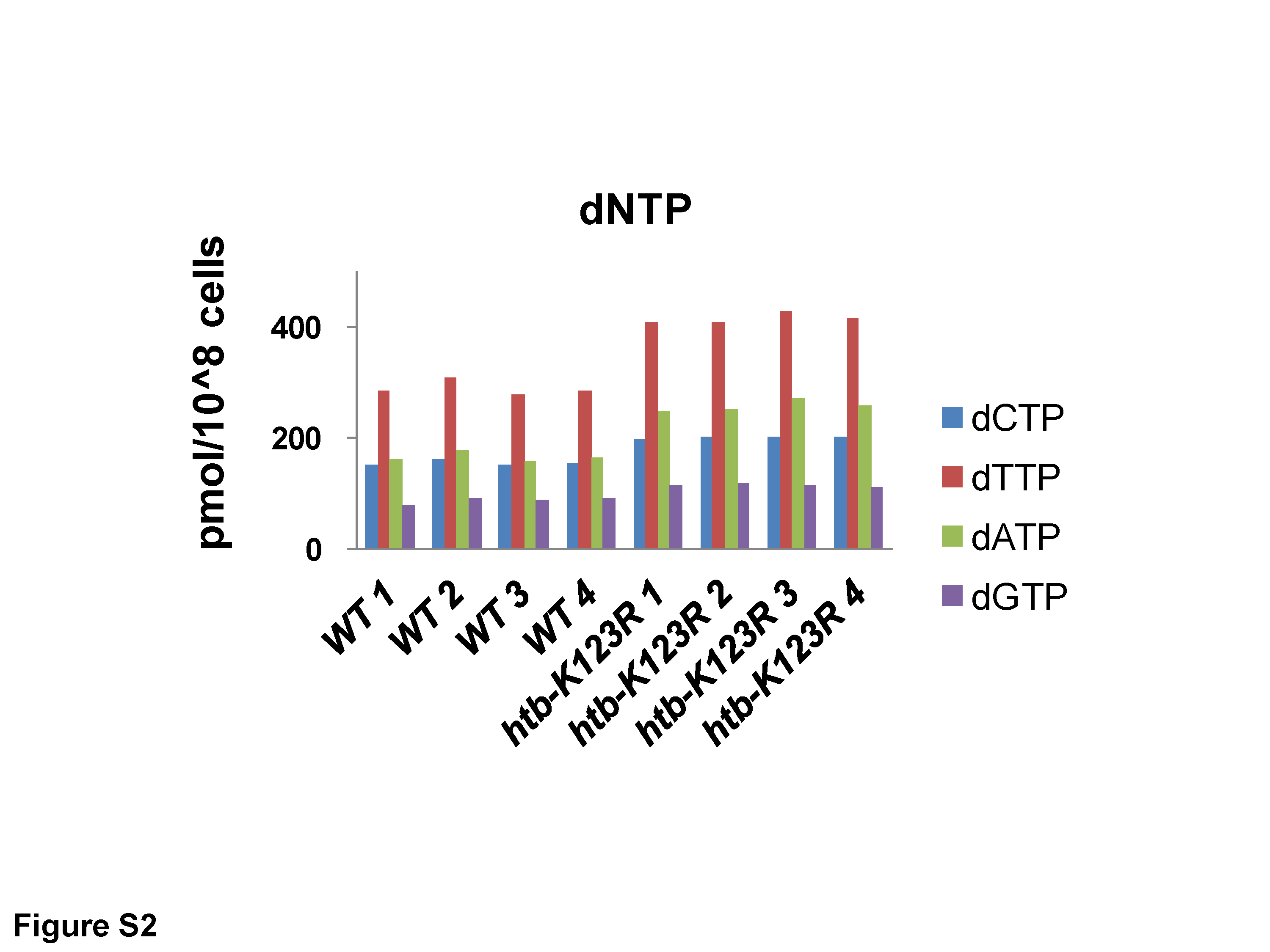

Supplement: Figure S2 — The size of each dNTP pool in exponentially-growing WT (CFK1419) and htb-K123R (CFK1421) cells. Four independent isogenic strains of each genotype were analyzed as described in the Materials and Methods. (TIFF) [file pgen.1004667.s002.tiff]

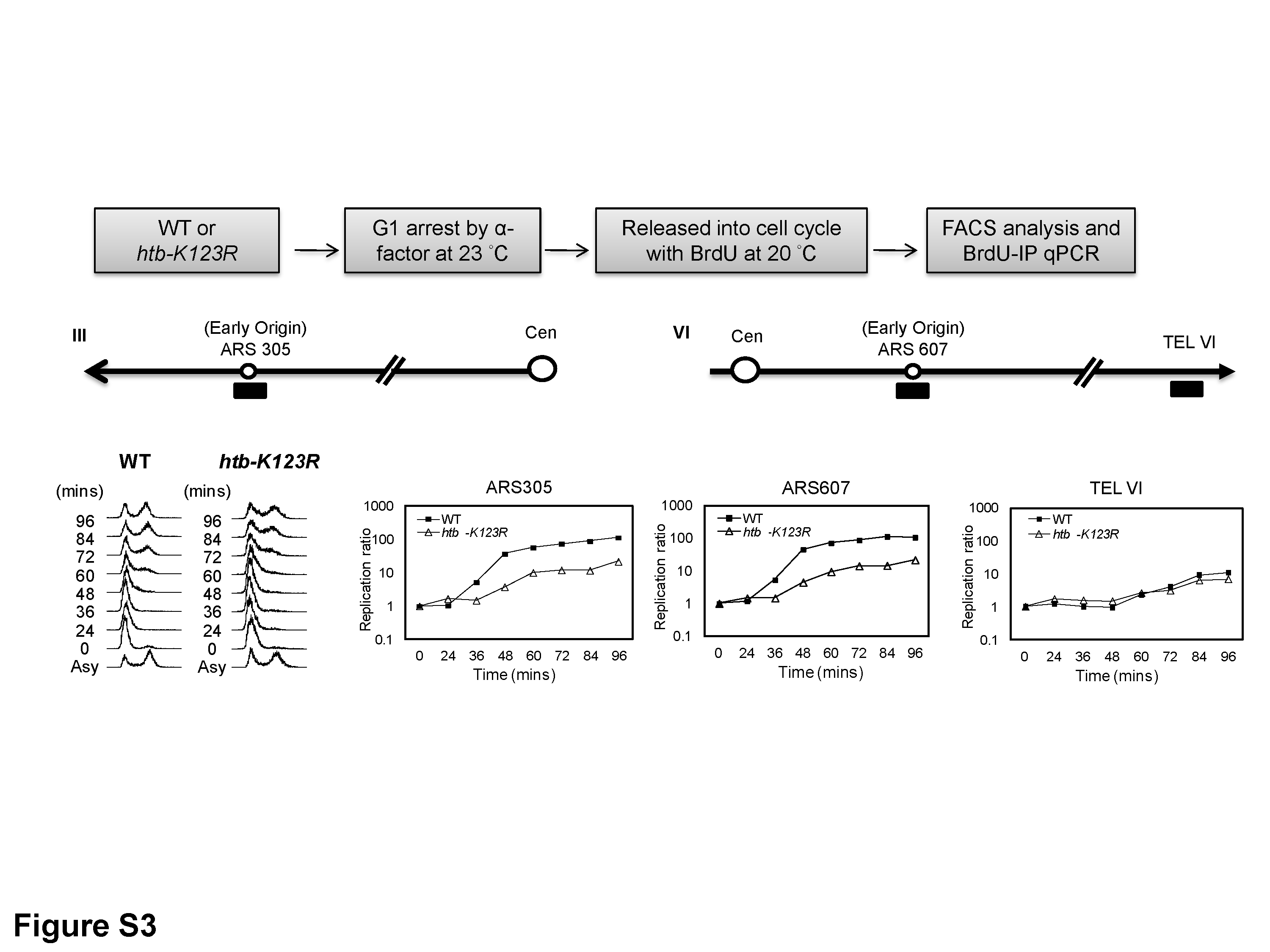

Supplement: Figure S3 — H2Bub is required for efficient origin firing. htb1-K123R mutants exhibit reduced BrdU incorporation during S phase. Cells were arrested at G1 using α-factor at 23°C, and released synchronously into S phase at 20°C in YPD supplemented with BrdU. Samples were collected at the indicated times and genomic DNA was then extracted. Monoclonal BrdU antibody was used to immunoprecipitate BrdU-incorporated DNA. DNA synthesis at replication origins (ARS305 and ARS607) or telomere was detected by quantitative-PCR. Cell cycle progression was monitored by FACS at 20°C under BrdU incorporation conditions. (TIFF) [file pgen.1004667.s003.tiff]

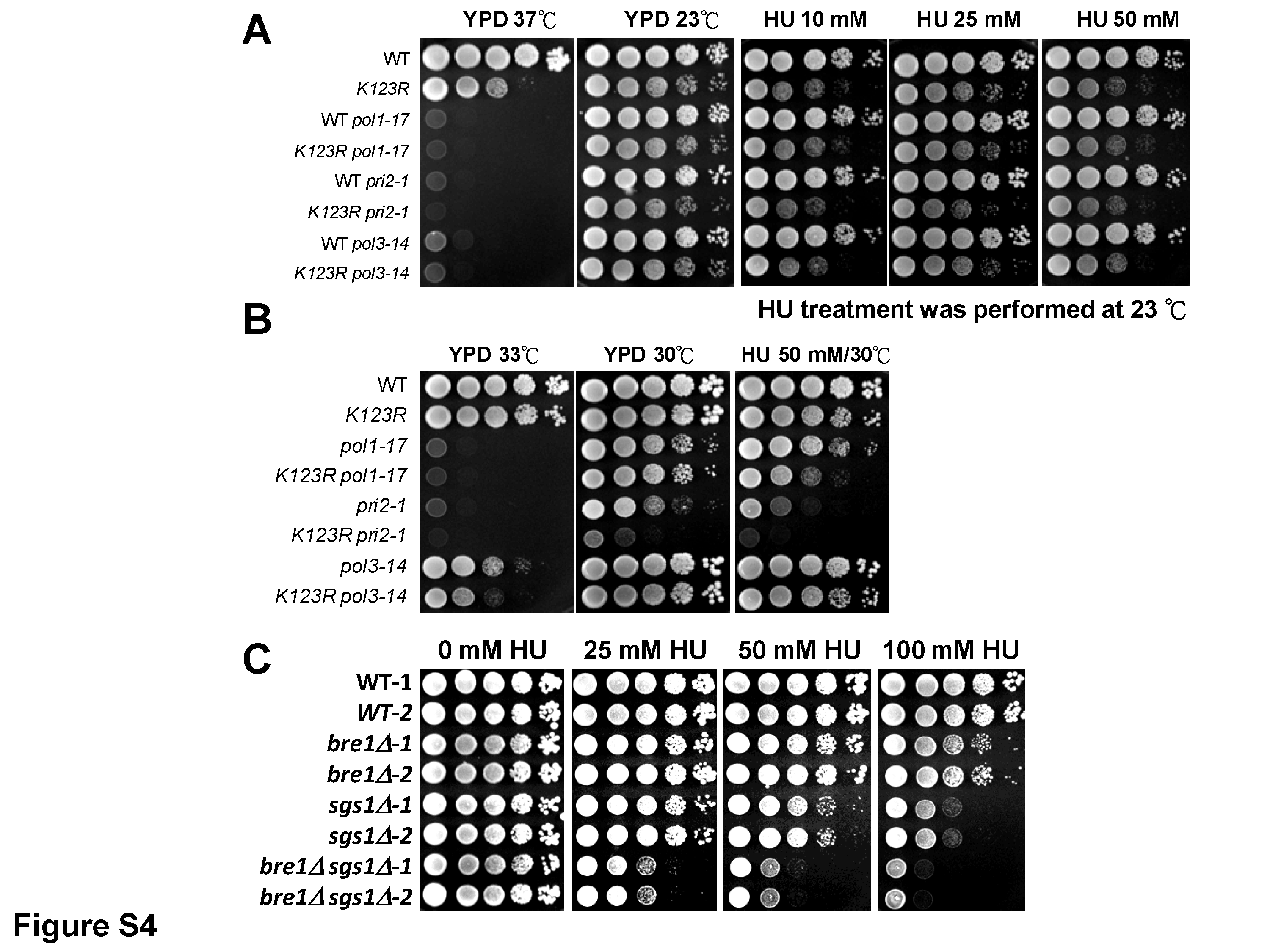

Supplement: Figure S4 — (A) The growth of htb-K123R and DNA polymerase ts double mutants are not affected by HU at the permissive temperature (23°C). Ten-fold serial dilutions of the indicated strains (WT (CFK1204), htb-K123R (CFK1231), pol1-17 (CFK1984), pol1-17 htb-K123R (CFK1986), pri2-1 (CFK1988), pri2-1 htb-K123R (CFK1990), pol3-14 (CFK1992) and pol3-14 htb-K123R (CFK1994)) were spotted onto YPD containing different doses of HU (0–50 mM) at 23°C for several days. Growth at the restrictive temperature (37°C) is presented as a control for ts mutants. (B) The genetic interaction between H2Bub and DNA pol1, pol3, or primase. Ten-fold serial dilutions of the indicated strains were spotted onto YPD, and growth was monitored at 33°C or 30°C, or under conditions of replication stress (50 mM HU) at 30°C. (C) The histone H2B ubiquitin E3 ligase Bre1 functions in parallel with the RecQ helicase Sgs1 under replication stress. Ten-fold serial dilutions of the indicated strains (WT (CFK1204), bre1Δ (CFK1443), sgs1Δ (CFK2371) and bre1Δ sgs1Δ (CFK2373)) were spotted onto YPD containing different doses of HU (0–100 mM) at 30°C. (TIFF) [file pgen.1004667.s004.tiff]

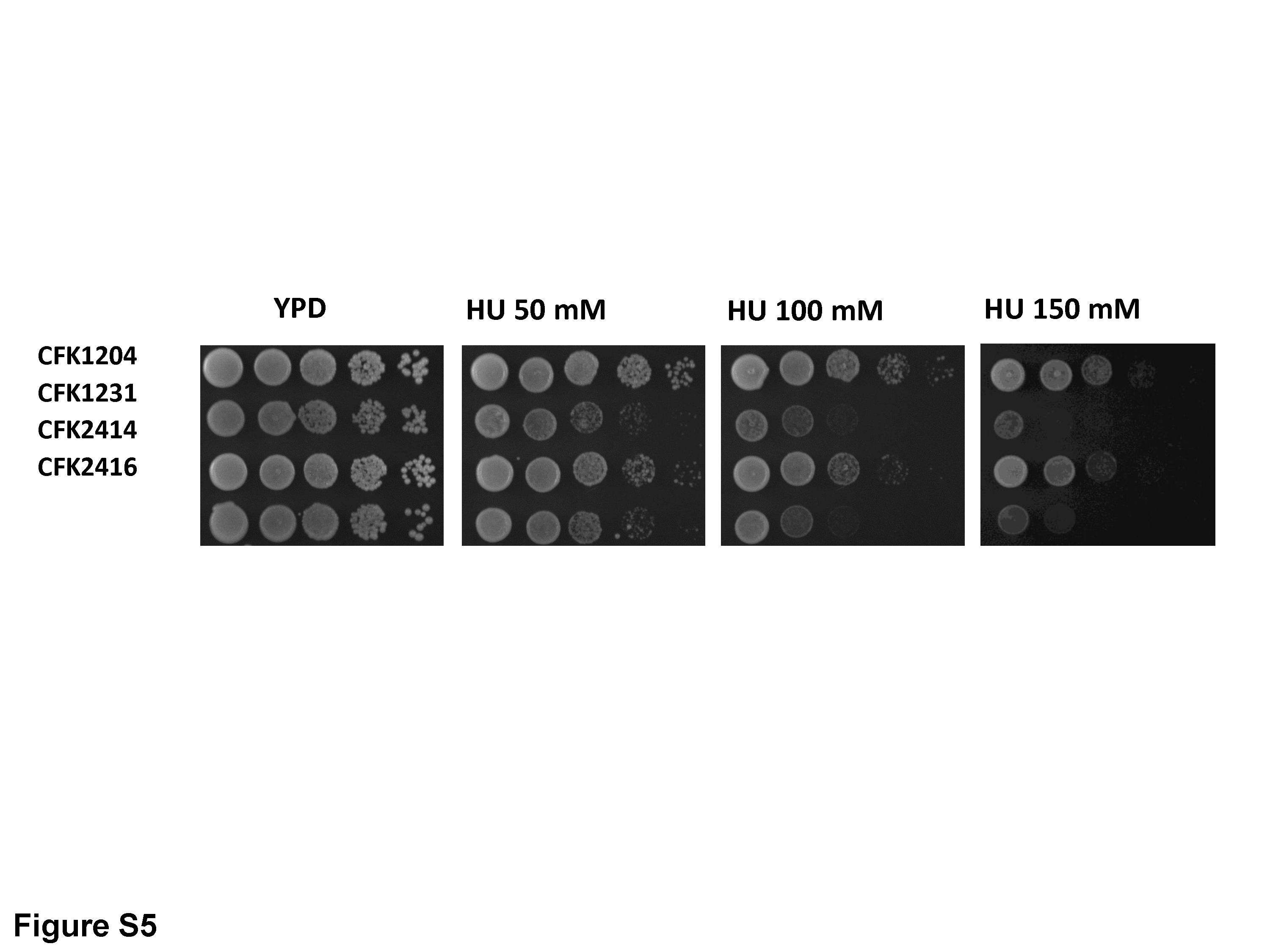

Supplement: Figure S5 — The growth of WT and htb-K123R cells of two different backgrounds under conditions of replication stress at 30°C. Ten-fold serial dilutions of the indicated strains (WT (CFK1204), htb-K123R (CFK1231), WT (CFK2414), and htb-K123R (CFK2416)) were spotted onto YPD containing different doses of HU (0–150 mM) for 2 days. Genotypes of the strains used: CFK1024: W303 hta1-htb1Δ hta2-htb2Δ CFK1031: W303 hta1-htb1Δ hta2-htb2Δ <pZS146-HTA1-Flag-htb1-K123R CEN HIS3> CFK2414: W303 CFK2416: W303 HTA1-htb1-K123R::NAT+ HTA2-htb2-K123R::HIS+. (TIFF) [file pgen.1004667.s005.tiff]

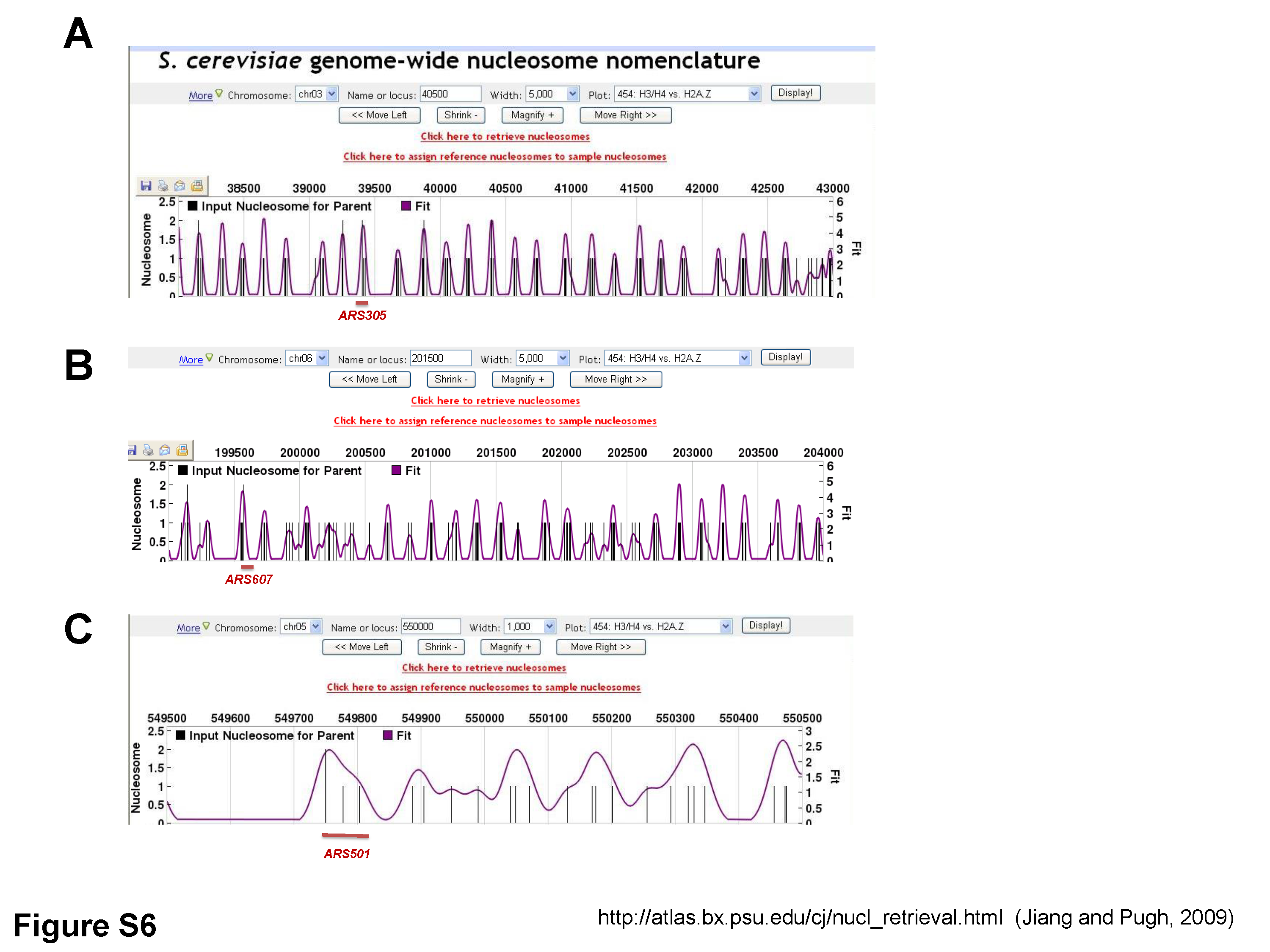

Supplement: Figure S6 — (A) A schematic description of the nucleosome position surrounding ARS305, and the primers used in Fig. 7A to amplify ARS305 for histone chromatin immunoprecipitation. ARS305 (nuc.) (39,349–39,455) primer sequence: (F): att tca gag cct tct ttg gag, (R): atg aaa ctg gac ata ttt gag gaa. (B) A schematic description of the nucleosome position surrounding ARS607, and the primers used in Fig. 7A to amplify ARS607 for histone chromatin immunoprecipitation. ARS607 (nuc.) (199,539–199,630) primer sequence: (F): aca cat tat tcg gca cag tag, (R): tcg cag tcc ata gaa gga g. (C) A schematic description of the nucleosome position surrounding ARS501, and the primers used in Fig. 7A to amplify ARS501 for histone chromatin immunoprecipitation. ARS501 (nuc.) (549,785–549,858) primer sequence: (F): ctcct catca tcatc cc, (R): cgtac actag cccgt tg. Image created using the following software available at the Penn State Genome Cartography Project http://atlas.bx.psu.edu/cj/nucl_retrieval.html [82] (TIFF) [file pgen.1004667.s006.tiff]
