# Supplementary material for: H2B Mono-ubiquitylation Facilitates Fork Stalling and Recovery during Replication Stress by Coordinating Rad53 Activation and Chromatin Assembly
Source: PLoS Genet. 2014 Oct 2;10(10):e1004667. doi: 10.1371/journal.pgen.1004667 (PMC4183429; doi:10.1371/journal.pgen.1004667)
Supplement: Table S1 — Yeast strains used in this study. (PDF) [file pgen.1004667.s007.pdf]

Table S1 Strain list

Strain	Genotype	Source
Y131	*MATa hta1-htb1Δ::LEU2 hta2-htb2Δ leu2-3,-112 his3-11,-15 trp1-1 ade2-1 can1-100 ura3-1 <pRS426-HTA1-HTB1>*	K. Robzyk[1]
CFK1065 (YS131)	*MATa hta1-htb1Δ hta2-htb2Δ leu2-3,-112 his3-11,-15 trp1-1 ade2-1 can 1-100 ura3-1 <pRS426-HTA1-HTB1>*	Y131<br>This study
CFK1179 (YS131 bar1Δ)	*MATa hta1-htb1Δ hta2-htb2Δ bar1Δ::LEU2 his3-11,-15 trp1-1 ade2-1 can 1-100 ura3-1 <pRS426-HTA1-HTB1>*	CFK1065<br>This study
CFK1204 (YCL001)	*MATa hta1-htb1Δ hta2-htb2Δ bar1Δ::LEU2 his3-11,-15 trp1-1 ade2-1 can1-100 ura3-1 <pZS145-HTA1-Flag-HTB1 CEN HIS3>*	CFK1179<br>This study
CFK1202	*MATa hta1-htb1Δ hta2-htb2Δ bar1Δ::LEU2 hir1Δ:: KAN+ his3-11,-15 trp1-1 ade2-1 can1-100 ura3-1 <pZS145-HTA1-Flag-HTB1 CEN HIS3>*	CFK1204<br>This study
CFK1206	*MATa hta1-htb1Δ hta2-htb2Δ barΔ1::LEU2 cac1Δ:: KAN+ his3-11,-15 trp1-1 ade2-1 can1-100 ura3-1 <pZS145-HTA1-Flag-HTB1 CEN HIS3>*	CFK1204<br>This study
CFK1208	*MATa hta1-htb1Δ hta2-htb2Δ bar1Δ::LEU2 asf1Δ:: KAN+ his3-11,-15 trp1-1 ade2-1 can1-100 ura3-1 <pZS145-HTA1-Flag-HTB1 CEN HIS3>*	CFK1204<br>This study
CFK1212	*MATa hta1-htb1Δ hta2-htb2Δ bar1Δ::LEU2 rtt109Δ:: KAN+ his3-11,-15 trp1-1 ade2-1 can1-100 ura3-1 <pZS145-HTA1-Flag-HTB1 CEN HIS3>*	CFK1204<br>This study
CFK1231 (YMW001)	*MATa hta1-htb1Δ hta2-htb2Δ bar1Δ::LEU2 his3-11,-15 trp1-1 ade2-1 can1-100 ura3-1 <pZS146-HTA1-Flag-htb1-K123R CEN HIS3>*	CFK1179<br>This study
CFK1233	*MATa hta1-htb1Δ hta2-htb2Δ bar1Δ::LEU2 asf1Δ:: KAN+ his3-11,-15 trp1-1 ade2-1 can1-100 ura3-1 <pZS146-HTA1-Flag-htb1-K123R CEN HIS3>*	CFK1231<br>This study
CFK1235	*MATa hta1-htb1Δ hta2-htb2Δ bar1Δ::LEU2 hir1Δ:: KAN+ his3-11,-15 trp1-1 ade2-1 can1-100 ura3-1 <pZS146-HTA1-Flag-htb1-K123R CEN HIS3>*	CFK1231<br>This study
CFK1237	*MATa hta1-htb1Δ hta2-htb2Δ bar1Δ::LEU2 cac1Δ:: KAN+ his3-11,-15 trp1-1 ade2-1 can1-100 ura3-1*	CFK1231<br>This study

	<pZS146-HTA1-Flag-htb1-K123R CEN HIS3>	
CFK1241	*MATa hta1-htb1Δ hta2-htb2Δ bar1Δ::LEU2 rtt109Δ:: KAN+ his3-11,-15 trp1-1 ade2-1 can1-100 ura3-1 <pZS146-HTA1-Flag-htb1-K123R CEN HIS3>*	CFK1231 This study
CFK1419	*MATa hta1-htb1Δ hta2-htb2Δ bar1Δ::LEU2 his3-11,-15 trp1-1 ade2-1 can1-100 ura3-1 <pZS145-HTA1-Flag-HTB1 CEN HIS3> <p404-BrdU-Inc-TRP1>*	CFK1204 This study
CFK1421	*MATa hta1-htb1Δ hta2-htb2Δ bar1Δ::LEU2 his3-11,-15 trp1-1 ade2-1 can1-100 ura3-1 <pZS146-HTA1-Flag-htb1-K123R CEN HIS3> <p404-BrdU-Inc-TRP1>*	CFK1231 This study
CFK1443	*MATa hta1-htb1Δ hta2-htb2Δ bar1Δ::LEU2 bre1Δ:: KAN+ his3-11,-15 trp1-1 ade2-1 can1-100 ura3-1 <pZS145-HTA1-Flag-HTB1 CEN HIS3>*	CFK1204 This study
CFK1444	*MATa hta1-htb1Δ hta2-htb2Δ bar1Δ::LEU2 mrc1Δ:: KAN+ his3-11,-15 trp1-1 ade2-1 can1-100 ura3-1 <pZS145-HTA1-Flag-HTB1 CEN HIS3>*	CFK1204 This study
CFK1447	*MATa hta1-htb1Δ hta2-htb2Δ bar1Δ::LEU2 sgs1Δ:: KAN+ his3-11,-15 trp1-1 ade2-1 can1-100 ura3-1 <pZS145-HTA1-Flag-HTB1 CEN HIS3>*	CFK1204 This study
CFK1450	*MATa hta1-htb1Δ hta2-htb2Δ bar1Δ::LEU2 mrc1Δ:: KAN+ his3-11,-15 trp1-1 ade2-1 can1-100 ura3-1 <pZS146-HTA1-Flag-htb1-K123R CEN HIS3>*	CFK1231 This study
CFK1453	*MATa hta1-htb1Δ hta2-htb2Δ bar1Δ::LEU2 sgs1Δ:: KAN+ his3-11,-15 trp1-1 ade2-1 can1-100 ura3-1 <pZS146-HTA1-Flag-htb1-K123R CEN HIS3>*	CFK1231 This study
CFK1481	*MATa hta1-htb1Δ hta2-htb2Δ bar1Δ::LEU2 sml1Δ:: KAN+ his3-11,-15 trp1-1 ade2-1 can1-100 ura3-1 <pZS145-HTA1-Flag-HTB1 CEN HIS3>*	CFK1204 This study
CFK1482	*MATa hta1-htb1Δ hta2-htb2Δ bar1Δ::LEU2 sml1Δ:: KAN+ his3-11,-15 trp1-1 ade2-1 can1-100 ura3-1 <pZS146-HTA1-Flag-htb1-K123R CEN HIS3>*	CFK1231 This study
CFK1764	*MATa hta1-htb1Δ hta2-htb2Δ bar1Δ::LEU2 sgs1-13myc::HIS3 pep4Δ::HPH+ trp1-1 ade2-1 can 1-100 ura3-1 <pRS414-HTA1-Flag-HTB1 CEN TRP>*	Y131 This study
CFK1765	*MATa hta1-htb1Δ hta2-htb2Δ bar1Δ::LEU2 sgs1-13myc::HIS3 pep4Δ::HPH+ trp1-1 ade2-1 can*	Y131 This study

	1-100 ura3-1 <pRS414-HTA1-Flag-htb1-K123R CEN TRP>	
CFK1984	*MATa hta1-htb1Δ hta2-htb2Δ bar1Δ::LEU2 pol1-17 his3-11,-15 trp1-1 ade2-1 can1-100 ura3-1 <pZS145-HTA1-Flag-HTB1 CEN HIS3>*	CFK1204<br>This study
CFK1986	*MATa hta1-htb1Δ hta2-htb2Δ bar1Δ::LEU2 pol1-17 his3-11,-15 trp1-1 ade2-1 can1-100 ura3-1 <pZS146-HTA1-Flag-htb1-K123R CEN HIS3>*	CFK1231<br>This study
CFK1988	*MATa hta1-htb1Δ hta2-htb2Δ bar1Δ::LEU2 pri2-1 his3-11,-15 trp1-1 ade2-1 can1-100 ura3-1 <pZS145-HTA1-Flag-HTB1 CEN HIS3>*	CFK1204<br>This study
CFK1990	*MATa hta1-htb1Δ hta2-htb2Δ bar1Δ::LEU2 pri2-1 his3-11,-15 trp1-1 ade2-1 can1-100 ura3-1 <pZS146-HTA1-Flag-htb1-K123R CEN HIS3>*	CFK1231<br>This study
CFK1992	*MATa hta1-htb1Δ hta2-htb2Δ bar1Δ::LEU2 pol3-14 his3-11,-15 trp1-1 ade2-1 can1-100 ura3-1 <pZS145-HTA1-Flag-HTB1 CEN HIS3>*	CFK1204<br>This study
CFK1994	*MATa hta1-htb1Δ hta2-htb2Δ bar1Δ::LEU2 pol3-14 his3-11,-15 trp1-1 ade2-1 can1-100 ura3-1 <pZS146-HTA1-Flag-htb1-K123R CEN HIS3>*	CFK1231<br>This study
CFK2000	*MATa hta1-htb1Δ hta2-htb2Δ bar1Δ::LEU2 his3-11,-15 trp1-1 ade2-1 can 1-100 ura3-1 <pRS426-HTA1-HTB1>*<br>*<pZS145-HTA1-Flag-HTB1 CEN HIS3>*	CFK1179<br>This study
CFK2002	*MATa hta1-htb1Δ hta2-htb2Δ bar1Δ::LEU2 his3-11,-15 trp1-1 ade2-1 can 1-100 ura3-1 <pRS426-HTA1-HTB1>*<br>*<pZS146-HTA1-Flag-htb1-K123R CEN HIS3>*	CFK1179<br>This study
CFK2004	*MATa hta1-htb1Δ hta2-htb2Δ bar1Δ::LEU2 pol2-11 his3-11,-15 trp1-1 ade2-1 can 1-100 ura3-1 <pRS426-HTA1-HTB1>*<br>*<pZS145-HTA1-Flag-HTB1 CEN HIS3>*	CFK1179<br>This study
CFK2006	*MATa hta1-htb1Δ hta2-htb2Δ bar1Δ::LEU2 pol2-11 his3-11,-15 trp1-1 ade2-1 can 1-100 ura3-1 <pRS426-HTA1-HTB1>*<br>*<pZS146-HTA1-Flag-htb1-K123R CEN HIS3>*	CFK1179<br>This study
CFK2346<br>(GA-4978)	*MATa Rad5+ mec1-100::LEU2(HIS+) ade2-1 ura3-1 his3-11 trp1-1 leu2-3,112 can 1-100*	W303 Rad5+<br>(Susan M.

		Gasser)[2]
CFK2347 (GA-1491)	*MATa rad53-11 ade2-1 ura3-1 his3-11 trp1-1 leu2-3,112 can 1-100*	W303 (NOY408-1b) (Susan M. Gasser)
CFK2351 (GA-180)	*MATa ade2-1 ura3-1 his3-11 trp1-1 leu2-3,112 can 1-100*	W303 (NOY408-1b) (Susan M. Gasser)
CFK2352 (GA-1981)	*MATa Rad5+ ade2-1 ura3-1 his3-11 trp1-1 leu2-3,112 can 1-100*	W303 Rad5+ (Susan M. Gasser)
CFK2356	*MATa Rad5+ mec1-100::LEU2(HIS+) HTA1-htb1-K123R::NAT+ HTA2-htb2-K123R::HIS+ ade2-1 ura3-1 his3-11 trp1-1 leu2-3,112 can 1-100*	GA4978 This study
CFK2358	*MATa rad53-11 HTA1-htb1-K123R::NAT+ HTA2-htb2-K123R::HIS+ ade2-1 ura3-1 his3-11 trp1-1 leu2-3,112 can 1-100*	GA1491 This study
CFK2371	*MATa hta1-htb1Δ hta2-htb2Δ bar1Δ::LEU2 sgs1Δ:: NAT+ his3-11,-15 trp1-1 ade2-1 can1-100 ura3-1 <pZS145-HTA1-Flag-HTB1 CEN HIS3>*	CFK1204 This study
CFK2373	*MATa hta1-htb1Δ hta2-htb2Δ bar1Δ::LEU2 sgs1Δ:: NAT+ bre1Δ:: KAN+ his3-11,-15 trp1-1 ade2-1 can1-100 ura3-1 <pZS145-HTA1-Flag-HTB1 CEN HIS3>*	CFK1204 This study
CFK2378	*MATa rad53-11 bre1Δ:: KAN+ ade2-1 ura3-1 his3-11 trp1-1 leu2-3,112 can 1-100*	GA1491 This study
CFK2414	*MATa ade2-1 ura3-1 his3-11 trp1-1 leu2-3,112 can 1-100*	W303 (NOY408-1b) This study
CFK2416	*MATa HTA1-htb1-K123R::NAT+ HTA2-htb2-K123R::HIS+ ade2-1 ura3-1 his3-11 trp1-1 leu2-3,112 can 1-100*	W303 (NOY408-1b) This study
YCL007	*MATa hta1-htb1Δ hta2-htb2Δ bar1Δ::LEU2 sgs1Δ:: KAN+ his3-11,-15 trp1-1 ade2-1 can1-100 ura3-1 <pZS145-HTA1-Flag-HTB1 CEN HIS3> <p404-BrdU-Inc-TRP1>*	CFK1447 This study
YCL008	*MATa hta1-htb1Δ hta2-htb2Δ bar1Δ::LEU2 sgs1Δ::*	CFK1453

	KAN+ his3-11,-15 trp1-1 ade2-1 can1-100 ura3-1 <pZS146-HTA1-Flag-htb1-K123R CEN HIS3> <p404-BrdU-Inc-TRP1>	This study
YMW069	*MATa hta1-htb1Δ hta2-htb2Δ bar1Δ::LEU2 dun1Δ:: KAN+ his3-11,-15 trp1-1 ade2-1 can1-100 ura3-1 <pZS145-HTA1-Flag-HTB1 CEN HIS3>*	CFK1204 This study
YMW072	*MATa hta1-htb1Δ hta2-htb2Δ bar1Δ::LEU2 dun1Δ:: KAN+ his3-11,-15 trp1-1 ade2-1 can1-100 ura3-1 <pZS146-HTA1-Flag-htb1-K123R CEN HIS3>*	CFK1231 This study
YMW093	*MATa bre1Δ:: KAN+ ade2-1 ura3-1 his3-11 trp1-1 leu2-3,112 can 1-100*	GA-180 This study
YMW095	*MATa Rad5+ mec1-100::LEU2(HIS+) bre1Δ:: KAN+ ade2-1 ura3-1 his3-11 trp1-1 leu2-3,112 can 1-100*	GA-4978 This study
YMW104	*MATa hta1-htb1Δ hta2-htb2Δ bar1Δ::LEU2 asf1-3HA his3-11,-15 trp1-1 ade2-1 can1-100 ura3-1 < pZS146-HTA1-Flag-htb1-K123R CEN HIS3>*	CFK1231 This study
YMW105	*MATa hta1-htb1Δ hta2-htb2Δ bar1Δ::LEU2 asf1-3HA his3-11,-15 trp1-1 ade2-1 can1-100 ura3-1 <pZS145-HTA1-Flag-HTB1 CEN HIS3>*	CFK1204 This study
YCL023	*MATa hta1-htb1Δ hta2-htb2Δ bar1Δ::LEU2 dun1Δ:: KAN+ his3-11,-15 trp1-1 ade2-1 can1-100 ura3-1 <pZS145-HTA1-Flag-HTB1 CEN HIS3> <p404-BrdU-Inc-TRP1>*	YMW069 This study
YCL025	*MATa hta1-htb1Δ hta2-htb2Δ bar1Δ::LEU2 dun1Δ:: KAN+ his3-11,-15 trp1-1 ade2-1 can1-100 ura3-1 <pZS146-HTA1-Flag-htb1-K123R CEN HIS3> <p404-BrdU-Inc-TRP1>*	YMW072 This study

1. Robzyk K, Recht J, Osley MA (2000) Rad6-dependent ubiquitination of histone H2B in yeast. Science 287: 501-504.
2. Duncker BP, Shimada K, Tsai-Pflugfelder M, Pasero P, Gasser SM (2002) An N-terminal domain of Dbf4p mediates interaction with both origin recognition complex (ORC) and Rad53p and can deregulate late origin firing. Proc Natl Acad Sci U S A 99: 16087-16092.
