# Supplementary material for: H2B Mono-ubiquitylation Facilitates Fork Stalling and Recovery during Replication Stress by Coordinating Rad53 Activation and Chromatin Assembly
Source: PLoS Genet. 2014 Oct 2;10(10):e1004667. doi: 10.1371/journal.pgen.1004667 (PMC4183429; doi:10.1371/journal.pgen.1004667)
Supplement: Table S2 — Plasmids used in this study. (PDF) [file pgen.1004667.s008.pdf]

Table S2 Plasmid list

Plasmids	Relevant genotype	Source
E1360 (pRS426)	HTA1- HTB1 CEN URA	K. Robzyk[1]
E1547 (pZS145)	HTA1-Flag-HTB1 CEN HIS3	M. Osley
E1118 (pZS146)	HTA1-Flag-htb1-K123R CEN HIS3	M. Osley
E1215 (p404)	BrdU-Inc-TRP1	O.M. Aparicio
E1356 (pJH1318)	YlP-pol1-17-URA	J. Haber
E1354 (pJH 1151)	YlPA16-pri2-1-URA	J. Haber
E1352	pRS306-pol2-11-URA	J. L. Campbell
E1358 (pJH1474)	pMJ14-pol3-14-URA	J. Haber
