# Supplementary material for: H2B Mono-ubiquitylation Facilitates Fork Stalling and Recovery during Replication Stress by Coordinating Rad53 Activation and Chromatin Assembly
Source: PLoS Genet. 2014 Oct 2;10(10):e1004667. doi: 10.1371/journal.pgen.1004667 (PMC4183429; doi:10.1371/journal.pgen.1004667)
Supplement: Table S3 — Primers used in this study. (PDF) [file pgen.1004667.s009.pdf]

**Table S3.** Primer sequences used in this study

| Name | Sequences | Applications |
|---|---|---|
| RNR1-F | 5’-TAC TGC GTG ACT TGG TTG-3’ | Gene expression |
| RNR1-R | 5’-TTG GTA AGC CTT GAA TGG AG-3’ | Gene expression |
| CDC21-F | 5’-ATT GTC TGA GCA AGG TGT-3’ | Gene expression |
| CDC21-R | 5’-TCC TAC TTT CCT ATC TTT GAA CCC-3’ | Gene expression |
| RNR3-F | 5’-CCA GCA TTC GTT GAG GT-3’ | Gene expression |
| RNR3-R | 5’-GTA ATG ACT TTA GCA ATC TCG TG-3’ | Gene expression |
| HUG1-F | 5’-CAA CCG TGT CAA CAA GAG T-3’ | Gene expression |
| HUG1-R | 5’-CAA TGT CAG AAA GAC CGC C-3’ | Gene expression |
| DDI2-F | 5’-CAG GTG GAA ATC AGG ACT ATG-3’ | Gene expression |
| DDI2-R | 5’-CAA GGT GGT AAT GTA GCC AG-3’ | Gene expression |
| ARS305-F | 5’-GGA CGA TAA TGT AAA TAG TTG TGG | ChIP (Sgs1) |
| ARS305-R | 5’-CAA AGA AGG CTC TGA AAT GC | ChIP (Sgs1) |
| ARS305 + 3.5K-F | 5’-TTG GGT GCC GCT ATT GAT | ChIP (Sgs1) |
| ARS305 + 3.5K-R | 5’-ACG GTT CAT TTG CTC TTC T | ChIP (Sgs1) |
| ARS607-F | 5’-GAG CTT TGT CTT GTT TAT ATT TAG TTA CG | ChIP (Sgs1) |
| ARS607-R | 5’-CTT ACG CTG GGT ATT TTT TTT TTG G | ChIP (Sgs1) |
| ARS607 + 4K-F | 5’-CAC ATT GTC AGT TGG TGG | ChIP (Sgs1) |
| ARS607 + 4K-R | 5’-AGT CGC TAA TGT CAC TAT GG | ChIP (Sgs1) |
| ARS305 (nuc.)-F | 5’-ATT TCA GAG CCT TCT TTG GAG | ChIP (H3) |
| ARS305 (nuc.)-R | 5’-ATG AAA CTG GAC ATA TTT GAG GAA | ChIP (H3) |
| ARS607 (nuc.)-F | 5’-ACA CAT TAT TCG GCA CAG TAG | ChIP (H3) |
| ARS607 (nuc.)-R | 5’-TCG CAG TCC ATA GAA GGA G | ChIP (H3) |
| ARS501 (nuc.)-F | 5’-CTCCT CATCA TCATC CC | ChIP (H3) |
| ARS501 (nuc.)-R | 5’-CGTAC ACTAG CCCGT TG | ChIP (H3) |
